# Pain, function and quality of life are impaired in adults undergoing periacetabular osteotomy (PAO) for hip dysplasia: a systematic review and meta-analysis

**DOI:** 10.1177/11207000231179610

**Published:** 2023-06-12

**Authors:** Michael JM O’Brien, Adam I Semciw, Inger Mechlenburg, Lisa CU Tønning, Chris JW Stewart, Joanne L Kemp

**Affiliations:** 1 Latrobe Sports Exercise Medicine Research Centre, School of Allied Health, Human Services and Sport, La Trobe University, Bundoora, Victoria, Australia; 2 MOG Sports Medicine, Melbourne Orthopaedic Group, Windsor, Victoria, Australia; 3 Department of Physiotherapy, Podiatry and Prosthetics and Orthotics, La Trobe University, Bundoora, Victoria, Australia; 4 Department of Orthopaedic Surgery, Aarhus University Hospital, Aarhus, Denmark; 5 Department of Clinical Medicine, Aarhus University, Aarhus, Denmark

**Keywords:** Developmental dysplasia of the hip, hip dysplasia, hip joint, periacetabular osteotomy, rehabilitation

## Abstract

**Background:**

Hip dysplasia is a common condition in active adults with hip pain that can lead to joint degeneration. Periacetabular osteotomy (PAO) is a common surgical treatment for hip dysplasia. The effect of this surgery on pain, function and quality of life (QOL) has not been systematically analysed.

**Purpose:**

In adults with hip dysplasia: (1) evaluate differences in pain, function and QOL in those undergoing PAO and healthy controls; (2) evaluate pre- to post-PAO changes in pain, function and QOL; (3) evaluate differences in pain, function and QOL in those with mild versus severe dysplasia, undergoing PAO; and (4) evaluate differences in pain, function and QOL in those having primary PAO versus those with previous hip arthroscopy.

**Methods:**

A comprehensive, reproducible search strategy was performed on 5 different databases. We included studies that assessed pain, function and QOL in adults undergoing PAO for hip dysplasia, using hip-specific patient reported outcomes measures.

**Results:**

From 5017 titles and abstracts screened, 62 studies were included. Meta-analysis showed PAO patients had worse outcomes pre- and post-PAO compared to healthy participants. Specifically, pain (standardised mean difference [SMD] 95% confidence interval [CI]): −4.05; −4.78 to −3.32), function (−2.81; −3.89 to −1.74), and QOL (−4.10; −4.43 to −3.77) were significantly poorer preoperatively.

Meta-analysis found patients experienced improvements following PAO. Pain improved from pre-surgery to 1-year (standardised paired difference [SPD] 1.35; 95% CI, 1.02–1.67) and 2 years postoperatively (1.35; 1.16–1.54). For function, the activities of daily living scores at 1 year (1.22; 1.09–1.35) and 2 years (1.06; 0.9–1.22) and QOL at 1 year (1.36; 1.22–1.5) and 2 years (1.3; 1.1–1.5) all improved. No difference was found between patients undergoing PAO with mild versus severe dysplasia.

**Conclusions:**

Before undergoing PAO surgery, adults with hip dysplasia have worse levels of pain, function and QOL compared to healthy participants. These levels improve following PAO, but do not reach the same level as their healthy participants.

**Registration:**

PROSPERO (CRD42020144748)

## Introduction

Developmental dysplasia of the hip (DDH) is a term that encompasses a spectrum of abnormal hip morphology involving the acetabulum and the proximal femur.^1,2^ Current notions describe a resultant instability of the hip joint with subsequent chondral degeneration and secondary osteoarthritis.^3^ It is thought that DDH is a risk factor of early-onset osteoarthritis.^3,4–8^ Amongst patients with mild degenerative change in their hip, those with DDH have almost 3 times the risk of progressing to end-stage osteoarthritis or total hip arthroplasty (THA), compared with normal morphology.^9^ In those with Tönnis grade 1 degenerative change, 1 in 3 patients received a THA within 10 years, compared to 1 in 5 shown in those with normal or femoroacetabular impingement (FAI) morphology.^9^ This implies that those with DDH are at increased risk of rapid degenerative change of their hip joint, once they develop early degenerative change.^9^

The international hip-related pain research network has identified acetabular dysplasia as one of the most common hip conditions in active adults presenting with hip pain.^10^ The true prevalence of DDH is difficult to ascertain as the condition can often be asymptomatic and there are inconsistencies regarding the diagnosis in the literature.^3,8,11^ Prevalence ranges from 1.7% to 20% in the general population.^7,11–14^ The traditional measure of DDH is the lateral centre-edge angle (LCEA) of Wiberg radiographically assessed from a weight-bearing anterioposterior (AP) pelvic view. A value <20° is defined as dysplasia, between 20° and 25° has been defined as borderline dysplasia, and 25–39° is defined as normal.^15^ It is unclear whether radiological severity affects outcomes. As the complex multi-directional nature of DDH has become better understood, the importance of utilising a combination of radiological and clinical findings has been recognised, though not agreed upon.^3,16,17^

Limited evidence suggests people suffering from DDH may experience pain, physical impairments, sporting limitations, and reduced quality of life (QOL).^18–20^ Surgical management options include arthroscopy, osteotomy, and THA.^3^ Hip arthroscopy is considered controversial and is often cautioned in patients with DDH due to conflicting outcomes,^3^ with 1 in 4 failures of hip arthroscopy occurring in patients with DDH as a primary or secondary diagnosis.^21^ Despite this, hip arthroscopy is commonly used in patients with DDH, and so requires evaluation. The most common surgical treatment used to address symptomatic DDH is periacetabular osteotomy (PAO).^22,23^ The procedure aims to preserve the native hip joint and delay the need for THA by medialising the hip joint centre, redistributing the high contact stresses from the acetabular rim to the entire articular surface, and transforming the dysplastic hip’s shear stresses across the articular cartilage into compressive stresses that are more favourable for cartilage longevity.^24–27^ Successful PAO surgery should not only improve structural abnormalities but also aim to improve pain, physical impairments, sporting limitations, and QOL. Several hip-specific patient-reported outcome measures (PROMs) exist to provide information on hip-related pain, function and QOL. However, there is limited systematic synthesis of PROMs, inhibiting our ability to confidently understand what these patients are experiencing. This affects our clinical approach and management, and our ability to truly appreciate the burden of this condition.

This systematic review aimed to, in adults with DDH: (1) evaluate differences in pain, function and QOL in those undergoing PAO and healthy controls; (2) evaluate pre- to postoperative changes in pain, function and QOL following PAO; (3) evaluate differences in pain, function and QOL in those with mild versus severe dysplasia, undergoing PAO; and (4) evaluate differences in pain, function and QOL in those having primary PAO versus those with previous hip arthroscopy.

## Methods

Study selection, eligibility criteria, data extraction, and statistical analysis were performed according to the Cochrane Collaboration guidelines.^28^ The systematic review was reported according to the preferred reporting guidelines for systematic reviews and meta-analysis (PRISMA) guidelines,^29^ and was registered on the Prospero international prospective register of systematic reviews (ID: CRD42020144748).

### Search strategy

A comprehensive, reproducible search strategy was performed on the following databases MEDLINE CINAHL, EMBASE, Sports Discuss, and PsychINFO from inception until 05 January 2021.

The search strategy was conducted by 2 reviewers (MO, AS) and using the following concepts:

(1) Humans with DDH aged ⩾15 years
(2) Periacetabular osteotomy
(3) Hip-specific patient-reported outcome measure

Synonyms were searched within concepts using ‘OR’ operator and searched between concepts using ‘AND’ operator.

For search strategy used see Supplemental Appendix 1.

All potential references were imported into Endnote X8 (Thomson Reuters, Carlsbad, CA, USA) and duplicates removed. All included studies were then uploaded into Covidence software (Veritas Health Innovation Ltd, Australia) for screening. Title, abstract and full text screening was conducted by 3 independent reviewers (MO (A–Z), CS (A–M), LR (N–Z). Any disagreements were resolved by a 4th independent reviewer (JK).

### Eligibility criteria

Studies were eligible for inclusion if they used a hip-specific patient-reported outcome measure (PROM) and were written in English. All quantitative observational study designs were considered eligible including randomised control trials, non-randomised controlled trials, case series, prospective or retrospective study designs.

### Participants/population

People aged 15 years and older with DDH undergoing PAO (based on the mean or median age of the study sample). Studies were ineligible if the PAO was undertaken in people with Cerebral Palsy, Down Syndrome or Charcot-Marie Tooth Disease populations.

### Intervention(s), exposure(s)

Studies utilising PAO surgery as primary intervention for DDH. The terms ‘Bernese Osteotomy’ and ‘Ganz Osteotomy’ were considered interchangeable with ‘Periacetabular Osteotomy’. Studies were ineligible if the PAO was reported to be a ‘rotational’ or ‘curved’ procedure as these procedures differ in surgical technique.

### Comparator(s)/control

Studies using sham treatment, no treatment or other treatment (e.g. THA or hip arthroscopy surgery) as the comparator/control treatment were included. We also included studies where no comparison group was present if they used 2 time-points (e.g. case series). In this instance, the pre-intervention time-point was considered the ‘comparison’.

### Outcomes

Primary outcomes of interest were hip-specific PROMs. These included: Hip disability and Osteoarthritis Outcome Score (HOOS), Western Ontario and McMaster universities osteoarthritis Index (WOMAC), the International Hip Outcome Tool (iHOT), the Copenhagen Hip and Groin Outcome Score (HAGOS), NonArthritic Hip Score (NAHS), and the Oxford Hip Score (OHS).

The HOOS is a PROM used for patients with reduced hip function with or without hip osteoarthritis, consisting of 5 subscales (pain, symptoms, activities of daily living, sport/recreation and QOL) with a 0–100 score for each, with 100 being the best possible result.^30^ In patients undergoing PAO the minimal clinically important difference (MCID) is 10.3 for pain, 10.2 for symptoms, 10.8 for activities of daily living, 12.6 for sport and recreation, and 11.2 for QOL.^31^ The HOOS has also shown adequate internal consistency and external validity.^31^

The WOMAC is a valid PROM for those with hip pain, consisting of 3 subscales (Pain, Stiffness, Function).^32^ Each subscale is summated to a maximum score of 20, 8, and 68, respectively. A lower score indicates a lower level of pain or symptoms. Typically, it has been used in older patients with degenerative joint disease but has also shown to be sensitive in a younger population following PAO.^33^ The minimal detectable change (MDC) has been reported as 5.51 for pain, 9.10 for function and 1.96 for stiffness,^31^ and the MCID has been reported as 10.8 for pain, 12.9 for stiffness, 10.8 for function, in patients with DDH undergoing PAO.^33^

The iHOT-33 is a PROM developed for younger active patients presenting with a variety of hip pathologies. Each score is out of 100, with 100 being the best score. It comprises of 33 questions relating to symptoms and functional limitations, sports and recreation activities, job-related concerns, and social, emotional and lifestyle concerns. The final score is then divided by 33. It has shown excellent validity and reliability with a minimal important change (MIC) of 6 and MDC ranging from 3.3 to 4.9 in those with hip pain.^34,35^

The HAGOS employs 6 subscales (symptoms, pain, function in daily living, function in sport and recreation, participation in physical activities, and QOL). Each subscale is scored from 0 to 100, with 100 being the best possible score. The HAGOS has also been used in patients following PAO and has been recently recommended one of the most appropriate PROMs to use in young and middle-aged active adults with hip-related pain.^36,37^

The NAHS was also developed for young active patients with higher demands and expectations.^38^ It consists of 20 items distributed in 4 domains of pain, mechanical symptoms, functional symptoms, and activity level. The NAHS has satisfactory reliability and fair validity.^34^

The OHS is a 12-question outcome measure assessing the patient’s hip pain and function.^39^ It generates a total score ranging from 0 to 48, where 48 indicates best possible result. The MIC for individual patients has been reported as 8, though this was in a population undergoing THA.^40^

Certain hip-specific PROMs were not eligible for inclusion in this systematic review due to their reported limitations in this population. The modified Harris Hip Score (mHHS) has been shown to have a lack of content validity and the presence of a ceiling effect.^34,41^ Similarly, the Hip Outcome Score (HOS) has also shown a ceiling effect and limited responsiveness following hip surgery.^41^ Therefore, studies that used the mHHS or the HOS as the primary PROM were not included. Studies using the Merle d’aubigne score, University of California Los Angeles activity-level rating score (UCLA), and visual analogue scale (VAS) were also excluded as these PROMs are not hip-specific. Studies using generic health-related QOL questionnaires were also not included as the purpose of this systematic review was to evaluate hip-specific outcome measures.

Studies were excluded if: (1) no full text was available; (2) the study was an animal study; or (3) the study was written in a language other than English.

### Quality evaluation

A modified version of the Downs and Black checklist was used to assess the quality of included studies. This modified version scores 18 potential criteria and has been used in other systematic reviews on hip pain.^42^ Studies were considered high quality with a score of >60%.^42^ Included studies were rated by 2 independent reviewers (MO, LR). Any disagreements between reviewers were discussed in a consensus meeting and an independent arbitrator (JK) was employed when consensus could not be met. Agreement between rates was determined using Cohen’s Kappa (K).

The Grades of Recommendation, Assessment, Development and Evaluation (GRADE) was applied to assess the quality of evidence for each meta-analysis.^43,44^ The overall GRADE certainty ratings include ‘very low’, ‘low’, ‘moderate’ and ‘high’. Observational data is initially graded at ‘low’ and can be increased or decreased for various reasons.^45^ Certainty can be rated up for (1) large magnitude of effect, (2) dose response gradient, (3) all residual confounding would decrease magnitude of effect. Certainty can be rated down for (1) risk of bias (if mean modified epidemiology appraisal instrument scored <60%), (2) imprecision (if upper or lower confidence interval [CI]) spanned a standardised mean difference [SMD] or standardised paired difference [SPD] of 0.5 in either direction), (3) inconsistency (if I^2^ was ⩾25%), (4) indirectness (if clinically heterogeneous) and (5) publication bias (for example, small studies that are industry-sponsored).

### Data extraction, synthesis and analyses

Data were extracted by 2 independent reviewers (MO, LR) into customised excel worksheets. The following data was extracted: author, year, country of origin, number of participants, demographic characteristics of participants (age, gender, body mass index [BMI], type of PAO), PROM scores, length of follow-up, and a summary of the findings was collated. Any discrepancies in data extraction were resolved by an independent reviewer (JK). A hierarchy of the different PROMs was decided on between authors to prioritise data extraction where more than one had been used, as recommended in the Cochrane guidelines.^28^ The order of the hierarchy was based on the established level of validity and reliability of the PROM for young people with hip pain, and applicability to people with DDH undergoing PAO. In order of selection, the hierarchy was HOOS, WOMAC, IHOT, HAGOS, NAHS, OHS. Where data was insufficient, authors were contacted and asked to provide missing data.

Studies were grouped according to design including: (1) between-group studies or (2) paired-data studies assessing change between pre- and post-PAO. If studies used a similar subscale, such as pain or QOL, at similar time-points then we performed meta-analysis using the random effects model. For between-group results this was done using Review Manager (RevMan) (Version 5.4.1 The Cochrane Collaboration, 2020), with a SMD and 95% CI for continuous data. The SMD is a summary statistic used to combine results from different studies that have measured similar outcomes but with different scales.^28^ For analysis of paired-data studies, a standardised paired difference (SPD) was calculated using R statistical software (version 4.0.4, Metafor package version 3.0-2). The SPD and 95% CI were calculated from the sample size, mean and SD of the difference between time-points. SMDs and SPDs of 0.2, 0.5 and 0.8 were interpreted as small, moderate and large effect sizes, respectively.^46^ Subgroup analyses were performed for specific time-points. Statistical heterogeneity across the pooled data was assessed using an I^2^ statistic, with 25% considered low, 50% moderate and 75% as high levels of heterogeneity.^47^

Where mean and SDs were not presented, we approximated mean scores from the median scores and SD from the range scores.^48^ Studies that only included total scores for outcomes that require subgroup scores, were not included in meta-analysis. Where patients had undergone 2 PAO surgeries, data was taken only for the first PAO. Participants were also excluded if it was easily identifiable that they had significant concomitant pathologies (e.g. Down syndrome, Charcot Marie Tooth Disease, septic arthritis). If postoperative data were not provided but change scores were, then the postoperative mean was calculated as the difference between the preoperative score and the change score. The preoperative standard deviation [SD] score was used as the postoperative SD score if this was unable to be imputed, as per the Cochrane guidelines.^33,49,50^

Where individual studies were not sufficiently homogenous to be included in a meta-analysis, a best evidence synthesis was used to provide an overall rating for the body of evidence.^51^ Grading of the best evidence synthesis was completed using previously published criteria.^42,52^ They were graded as strong (⩾2 studies with low risk of bias and ⩾75% agreement), moderate (⩾2 studies including at least 1 low risk of bias and ⩾75% agreement), limited (⩾1 moderate/high risk of bias studies, with ⩾75% agreement, or 1 low risk of bias study), conflicting (inconsistent findings <75% agreement), or no evidence.

## Results

### Search strategy

**Figure 1.**
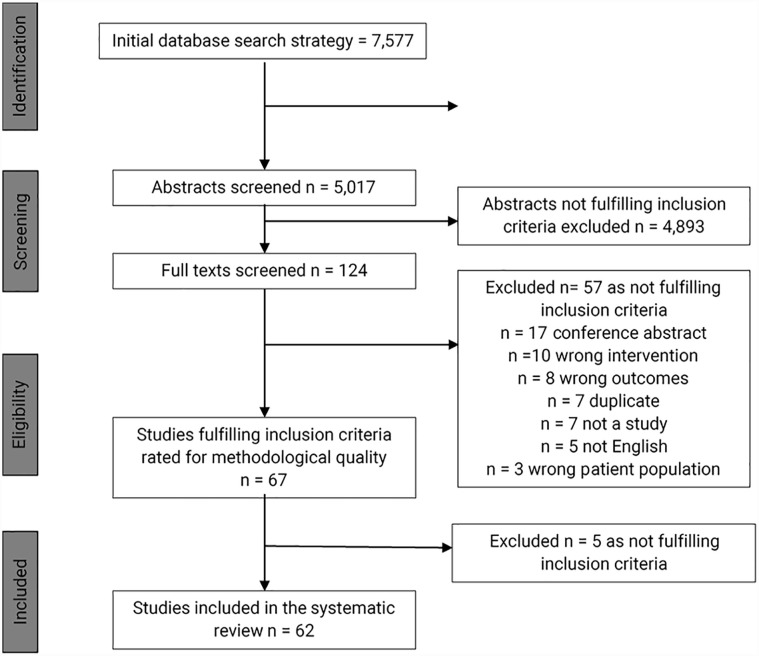
Preferred Reporting Items for Systematic Reviews and Meta-Analysis (PRISMA) flow diagram.

The search yielded 5017 titles and abstracts for screening. 124 full-text studies were screened, and 62 studies were excluded. 62 studies fulfilled the inclusion criteria and were included in this systematic review. An overview of the study identification process is provided in Figure 1.

### Methodological quality

Supplemental Appendix 2 contains the results of risk of bias assessment using the modified Downs & Black checklist. Initial agreement between quality assessors was moderate (K = 0.546).^53^ The methodological quality scores ranged from 39% to 94%,^54,55^ with an overall mean (SD) rating of 71% (11.6%). Of the included studies, 61 (98%) clearly described their aims or hypothesis and 60 (97%) outlined their main outcomes in the introduction or methods section. 17 studies (27%) stated if the main outcome measures used were valid and reliable, and only 8 studies (13%) provided characteristics of patients lost to follow-up.

### Participants

The 62 studies included 8222 participants, with 6852 of these participants undergoing PAO. A proportion of these participants represent data-points that were published on multiple occasions. Sample sizes of the PAO groups ranged from 16 patients to 599 patients.^56,57^ The mean (SD) ages for patients in the included studies ranged from 17 years to 45 years.^58,59^ 26 studies were single cohort studies which assessed PROM preoperatively and postoperatively, ^29,30,32,35,48,58,60–80^ 3 studies compared those having PAO as a first-time surgery with those who have had previous arthroscopy,^81–83^ and 3 studies compared those having PAO with healthy controls.^19,84,85^

### Outcome measures

17 studies used the HOOS, 33 studies used the WOMAC, 5 studies used the iHOT, 4 studies used the HAGOS, 3 studies used the NAHS, and no studies used the OHS. Study details are contained in Table 1. When a study used more than 1 of these questionnaires, only data from the highest-ranking PROM in our hierarchy was used.

**Table 1.** Summary of included studies.

Author (year) Study type	Sample size/comparison Sex (Female %)/comparison Age mean (years)/comparison BMI mean (kg/m^2^)/comparison	Quality Appraisal (using modified Downs & Black)	Population/Inclusion criteria	Exclusion criteria	PROM data extracted	Comparison Group	Timepoints	SMD Between Group Positive value = PAO group or first comparison favoured	SPD Paired Data Positive value = Improvement from baseline to follow-up.
Beaulé et al.^60^ (2015) Retrospective case series	*n =* 67 69% 32y(R14–54) 26kg/m^2^	12/16	PAO patients. Surgical criteria: acetabular dysplasia with a CEA <25°, hip pain for ⩾1 year, and failure of non-surgical management (medications and physiotherapy).	Surgical exclusion for PAO: (1) Tönnis grade 2 (2) lack of congruity (3) age >50 years.	WOMAC Pain	NA	Pre-op, 1 year post-op	NA	0.99(0.70–1.29)^a^
					WOMAC Stiffness				0.86(0.58–1.14)^a^
					WOMAC Function				0.76(0.48–1.03)^a^
Belzile et al.^86^ (2016) Retrospective cohort Study	*n =* 144/*n =* 144 82%/82% 27y(R15 – 46)/27y(R14 – 46) 24kg/m^2^/24kg/m^2^	11/18	Patients undergoing PAO for DDH. Comparison group: patients treated for FAI	LCPD, neuromuscular disorders, bilateral procedures, joint space narrowing of >2 mm, unavailable for 2-year follow-up and concomitant femoral corrective osteotomy	WOMAC Pain	FAI (open and arthroscopic surgery)	Pre-op, 1 year post-op, 2 years post-op	**Pre-op:** 0.21(-0.02–0.44)^b^ **1-year:** 0.84(0.6–1.08)^b^ **2-years:** 0.09(-0.14–0.32)^b^	NA
					WOMAC Stiffness			**Pre-op:** 0.30(0.07–0.53)^b^ **1-year:** 0.54(0.3–0.77)^b^ **2-years:** 0.09(-0.14–0.32)^b^	
					WOMAC Function			**Pre-op:** 0.26(0.03–0.5)^b^ **1-year:** 0.4(0.17–0.64)^b^ **2-years:** -0.01(-0.24–0.22^b^	
Biederman et al.^87^ (2008) Retrospective case series	*n =* 50 72% 27y(R12 – 44) NR	12/18	PAO with ⩾2 years follow-up	NR	WOMAC	NA	Mean 7.4 years post-op		Not estimable^b^
Bogunovic et al.^61^ (2014) Retrospective cohort study	*n =* 36 58% 25y(R15 – 45) 24 kg/m^2^ (3.5)	10/16	PAO with preoperative UCLA score of ⩾7 and minimum of 18 months follow-up	NR	HOOS	NA	Pre-op and mean 33 months post-op	NA	Not-estimable but favoured follow-up^b^
Boje et al.^30^ (2019) Retrospective cohort study	*n =* 321 88% 31y(R14-49) 23 kg/m^2^(R16 – 34)	10/16	Patients undergoing PAO for DDH, who completed pre-operative questionnaire. Surgical indications: CEA <25°, persistent hip pain, reduced walking distance, hip congruence, Tönnis OA Grade 0–1, hip flexion >110° and internal hip rotation >15°	Incomplete PROMs, underwent PAO due–other diagnoses DDH, underwent reverse PAO or femoral osteotomy, in patients operated bilaterally, the second operated hip was excluded	HOOS Pain	NA	Pre-op and 2 years post-op	NA	1.29(1.15–1.44)^a^
					HOOS Symptoms				0.95(0.82–1.08)^a^
					HOOS ADL				1.11(0.98–1.23)^a^
					HOOS Sport				1.32(0.88–1.75)^a^
					HOOS QOL				1.31(1.04–1.57)^a^
Brusalis et al.^62^ (2020) Retrospective study	*n =* 25 100% 27y(7) NR	12/18	PAO patients who had ⩾1 prior hip arthroscopy on ipsilateral hip, a preoperative LCEA of ⩽24°, a Tonnis angle of <10°, and ⩾6 months of follow-up clinical outcomes data Surgical indications: PAO performed if failed hip arthroscopy (persistent or recurrent pain within 5 years of arthroscopy	Incomplete radiographic data	iHOT-33	NA	Pre-op and mean 22 months	NA	0.88(0.42–1.34)^b^
Cates et al.^63^ (2019) Prospective cohort study	*n =* 23 83% 25y(7) 25kg/m^2^ (6)	10/16	Patients undergoing PAO for DDH and retroversion	NR	HOOS	NA	Pre-op, 1 year and 2 years post-op	NA	Not estimable but favoured follow-up^b^
Clohisy et al.^64^ (2017) Prospective cohort study	*n =* 391 79% 25y(10) 25kg/m^2^	12/16	Patients undergoing PAO for DDH	PAO for another diagnosis and revision PAO	HOOS	NA	Pre-op and mean 2.6 years post-op	NA	Not estimable but favoured follow-up^b^
Dahl et al.^88^ (2014) Cross-sectional study	*n =* 116 NR NR NR	13/16	Patients undergoing PAO	NR	WOMAC	NA	7 years post-op	NA	Not estimable^b^
Davidson et al.^33^ (2011) Retrospective cohort study	*n =* 83 17% 27y(R17-42) NR	8/16	PAO patients with completed WOMAC and SF-36 questionnaires	Non-completed questionnaires	WOMAC Pain	NA	Pre-op and 2 years post-op	NA	1.34(1.04–1.64)^a^
					WOMAC Stiffness	NA			Not estimable but favoured follow-up^b^
					WOMAC Function	NA			Not estimable but favoured follow-up^b^
Domb et al.^65^ (2015) Retrospective case series	*n =* 17 82% 24y(7) 24kg/m^2^(5)	9/16	Patients undergoing hip arthroscopy and PAO	NR	NAHS	NA	Pre-op and 2 years post-op	NA	Not estimable but favoured follow-up^b^
Duncan et al.^89^ (2015) Retrospective cohort study	*n =* 180 77% 26y 24 kg/m^2^	11/16	Patients undergoing PAO for symptomatic acetabular deformity	Perthes-like deformities, acetabular retroversion, no available digital radiographs, ipsilateral osteotomy	HOOS	NA	Males vs. females	NA	Not estimable^b^
Edelstein et al.^66^ (2021) Retrospective cohort study	*n =* 67 93% 29y(10) 24 kg/m^2^(4)	11/16	Patients undergoing concurrent arthroscopy and PAO Surgical criteria: Hip pain affecting daily function, persistent following 3 months of nonsurgical treatments, LCEA <20° on radiographs without degenerative changes.	Diagnoses other than DDH. Associated neuromuscular disease, LCPD, SCFE, post-traumatic deformity, and isolated acetabular retroversion	WOMAC Pain	NA	Pre-op and 6.5 years post-op	NA	1.42(1.08–1.76)^a^
Garbuz et al.^59^ (2008) Cross-sectional cohort study	*n =* 28/34 90%/88% 45y/47y NR	9/18	DDH with minimal or no osteoarthritis (Tönnis grade 0 or 1), age >40 years, and 2-year follow-up data	NR	WOMAC	THA	4 years post-op	Not estimable but results favoured THA group^b^	NA
Goronzy et al.^90^ (2017) Retrospective cohort study	*n =* 32/42 100%/75% 27y(11)/29y(10) 23(4) kg/m^2^/24 kg/m^2^(4)	12/18	Patients undergoing PAO for DDH. Surgical criteria: decreased LCEA with hip pain lasting ⩾6 months not responding adequately to conservative therapy	Surgical PAO contraindications: advanced radiographic OA (Kellgren-Lawrence Grade 3 & 4), joint space incongruency on radiographs, or patient age >50 years	WOMAC Pain	Isolated PAO / PAO+CAM	Pre-op and 31–102 months post-op	**Pre-op:** -0.05(-0.51–0.41)^b^ 6 years: 0.0(-0.46–0.46)^b^	NA
					WOMAC Stiffness			**Pre-op:** 0.15(-0.31–0.61)^b^ 6 years: 0.0(-0.46–0.46)^b^	
					WOMAC Function			**Pre-op:** 0.16(-0.3–0.62)^b^ 6 years: 0.07(-0.39–0.53) ^b^	
Goronzy et al.^67^ (2020) Case series	*n =* 86 82% 27y(10) 24 kg/m^2^(4)	12/16	Patients undergoing isolated PAO for DDH Surgical criteria: decreased LCEA with hip pain lasting ⩾6 months not responding adequately–conservative therapy	Surgical PAO contraindications: advanced radiographic OA (Kellgren-Lawrence Grade 3 & 4), joint space incongruency on radiographs, or patient age >50 years. Minors.	WOMAC	NA	Pre-op and mean 62 months post-op	NA	Not estimable^b^
Grammatopoulos et al.^91^ (2018) Retrospective case series	*n =* 244 84% 26y(10) 24 kg/m^2^(4)	11/18	Patients undergoing PAO surgery	LCPD, SCFE, neuromuscular conditions, skeletal dysplasia, and no 2-year+ follow-up data	HOOS	NA	Pre-op and 4 years post-op	NA	Not estimable^b^
Grammatopoulos et al.^92^ (2016) Retrospective case series	*n =* 57 86% 25y(7) 24 kg/m^2^(3)	13/16	Patients undergoing PAO for symptomatic DDH, developmentally mature hip without evidence of major joint incongruence or subluxation.	NR	WOMAC	NA	Mean 8 years post-op	NA	Not estimable^b^
Hartig-Andreasen et al.^93^ (2015) Prospective cohort study	*n =* 90 88% 34y(R15-59) NR	14/18	Patients following PAO. Surgical indications: persistent hip pain, a CEA of <25°, pelvic bone maturity, IR>15°, hip flexion <110° and Tönnis grade of 0 or 1.	Multiple complaints from several joints, failure–show up at 2-year follow-up.	WOMAC Pain	Hip Arthroscopy after PAO	Pre-op and 2 years post-op	0.77(0.3–1.24)^b^	NA
					WOMAC Stiffness			0.6(0.13–1.06)^b^	
					WOMAC Function			0.65(0.19–1.12)^b^	
Hartig-Andreasen et al.^94^ (2012) Cross-sectional study	*n =* 316 72% 33.9y(R13– 61) 24 kg/m^2^(R15–37)	13/16	Patients undergoing PAO. Surgical indications: persistent hip pain, CEA of <25°, pelvic bone maturity, absence of hip subluxation, IR of >15°, and hip flexion >110°.	Incomplete follow-up data or death. PAO contraindications: OA, reduced ROM, lack of hip congruence.	WOMAC	NA	Post-op (R4–12 years)	Not estimable^b^	NA
Heyworth et al.^95^ (2016) Retrospective case series	*n =* 46 88% 26y(R13–41) NR	11/16	Aged 10–45 years at the time of surgery, minimum UCLA-AS score of 8/10, self-reported sport participation, and completed a hip questionnaire before surgery and ⩾1-year post-op. Surgical indications: hip pain secondary–DDH with LCEA of <20°	Underlying neuromuscular disease, incomplete questionnaires, or not an athlete.	HOOS	NA	Pre-op and 3 years post-op	NA	Not estimable^b^
Hingsammer et al.^68^ (2015) Prospective cohort study	*n =* 37 92% 26y(9) NR	10/16	Symptomatic DDH with a LCEA of <20°	Hip flexion of <90°, Tönnis grade of >1, neuromuscular and chromosomal disorders, and an incongruous hip joint on radiographs	WOMAC	NA	Pre-op, 1 year and 2 years post-op	NA	Not estimable but favoured follow-up^b^
Hsieh et al.^96^ (2009) Retrospective case-control study	*n =* 31/31 84%/84% 32y(R29–52) NR	11/18	Patients who had undergone both PAO and THA on the contralateral side. Surgical indication: progressive hip pain.	THA performed at a different institution, lost–follow-up before two years after operation, previous hip surgery	WOMAC	THA	Post-op (mean 7, R3–10 years)	Not estimable^b^	NA
Jacobsen et al.^19^ (2014) Prospective cohort study	*n =* 32/32 81%/81% 34y(R18–53)/33y(R18–54) 22 kg/m^2^/22 kg/m^2^	14/18	Diagnosis of DDH, planned pelvis operation, Tönnis OA grade 0–1, aged between 18–69 years	LCPD or epiphysiolysis, previous operations due–a herniated disc, joint preservation, or alloplastic surgery at the hip, knee or ankle region, or neurological or rheumatological disease	HAGOS Pain	Healthy Controls	Pre-op and 6 months post-op	**Pre-op:** -3.31(-4.08–-2.54)^a^ **6 months:** -2.19(-2.83–-1.54)^a^ **1 year:** -1.56(-2.14–-0.98)^a^	NA
					HAGOS Symptoms		**Pre-op:** -3.42(-4.21–-2.64)^a^ 6 months: -2.03(-2.66–-1.4)^a^ 1 year: -1.71(-2.30–-1.12)^a^		
					HAGOS ADL		**Pre-op:** -2.23(-2.86–-1.6)^a^ 6 months: -1.44(-2.01–-0.87)^a^ 1 year: -1.12(-1.67–-0.58)^a^		
					HAGOS Sport		**Pre-op:** -3.37(-4.15–-2.6)^a^ 6 months: -2.30(-2.96–-1.64)^a^ 1 year: -1.76(-2.36–-1.16)^a^		
					HAGOS Participation		**Pre-op:** -2.88(-3.59–-2.17)^b^ 6 months: -2.04(-2.67–-1.4)^b^ 1-year: -1.48(-2.05–-0.91)^b^		
					HAGOS QOL		**Pre-op:** -3.83(-4.67–-2.99)^a^ 6 months: -2.48(-3.17–-1.80)^a^ 1 year: -1.81(-2.41–-1.21)^a^		
Jacobsen et al.^36^ (2019) Prospective case series	*n =* 82 87% 30y(9) 23kg/m^2^(3)	13/16	Surgical indications: LCEA of <25°, groin pain >3 months, and scheduled for PAO, <45 years, with BMI <30, >110° of hip flexion, and with Tönnis grade <2	Patients with comorbidities and previous surgical interventions affecting their hip function	HAGOS Pain	NA	Pre-op and 1 year post-op	NA	1.52(1.20–1.83)^a^
					HAGOS Symptoms				1.04(0.77–1.31)^a^
					HAGOS ADL				1.27(0.98–1.56)^a^
					HAGOS Sport				1.29(1.01–1.57)^a^
					HAGOS QOL				1.28(0.99–1.57)^a^
Kain et al.^81^ (2011) Retrospective cohort study	*n =* 34/17 100%/100% 31y(10)/31y(10) NR	11/16	PAO only with MRI evidence of labral pathology prior–surgery / Initial arthroscopy for labral tear but eventual PAO	NR	WOMAC Pain	Initial arthroscopy for labral tear but eventual PAO	Pre-op and post-op (unspecified)	**Pre-op:** -0.22(-0.91–0.48)^a^ Post-op: -0.40(-1.10–0.30)^b^	NA
					WOMAC Stiffness			**Pre-op:** -0.77(-1.49–-0.05)^a^ Post-op: -0.37(-1.07–0.33)^b^	
					WOMAC Function			**Pre-op:** -0.52(-1.24–0.17)^a^ Post-op: -0.51(-1.22–0.19)^b^	
Karam et al.^69^ (2011) Prospective case series	*n =* 33 82% 29y(R16–50) 27kg/m^2^(R19–38)	10/16	PAO for symptomatic DDH	NR	WOMAC	NA	Pre-op and 1 year post-op	NA	Not estimable but favoured follow-up^b^
Khan et al.^49^ (2017) Prospective longitudinal cohort study	*n =* 151 90% 32y(R15–56) NR	12/16	Patients undergoing PAO. Surgical indications: symptomatic DDH that had failed non-surgical treatment with a CEA <25°, AI >10° and a congruent hip joint	Surgery for acetabular retroversion.	NAHS	NA	Pre-op and 3 years post-op	NA	Not estimable but favoured follow-up^b^
Kralj et al.^70^ (2005) Cross-sectional study	*n =* 26 85% 30y(R18–50) NR	9/16	Patients undergoing PAO for DDH	Missing radiographs	WOMAC	NA	Pre-op and mean 12 years (R7–15)	NA	Not estimable^b^
Larsen et al.^57^ (2020) Retrospective study	*n =* 599 85% 32y(R13–59) NR	14/16	PAO surgical indications: symptomatic DDH with persistent hip pain and reduced function, LCEA < 25°, pelvic bone maturity, absence of hip subluxation, IR >15°, and hip flexion >110°, Tönnis OA grade 0, BMI ⩽ 25 and age ⩽ 45 years.	Reverse PAO, femoral osteotomy, persons without a Danish civil registration number, LCPD, and congenital hip dislocation. Surgical contraindications: OA, reduced ROM indicating joint degeneration, lack of hip congruence, BMI > 30 kg/m^2^	HOOS	NA	Pre-op, 6 months, 2 years, 5 years and 10 years	NA	Not estimable but favoured follow-up^b^
Li et al.^71^ (2020) Retrospective study	*n =* 220 84% 28y(8) 25kg/m^2^(4)	10/16	Patients undergoing PAO symptomatic DDH who did not respond–nonoperative treatment	Patients <18 years, history of ipsilateral hip surgery	HOOS Pain	NA	Pre-op and mean 1.5 years (R1–2.9) post-op	NA	1.74(1.33–2.16)^b^
					HOOS Symptoms				1.28(0.93–1.64)^b^
					HOOS ADL				1.63(1.23–2.02)^b^
					HOOS Sport				1.85(1.41–2.28)^b^
					HOOS QOL				1.51(1.13–1.89)^b^
Maeckelbergh et al.^84^ (2018) Cross-sectional study	*n =* 42/963 76%/66% 27y(R14–50)/NR NR/24kg/m^2^(R11–38)	13/18	PAO for symptomatic DDH	Major intra- or postoperative complications. Incomplete or absent PROMs	HOOS Pain	Healthy Controls	Pre-op and mean 2.6 years (R1–5)	**Pre-op:** -4.23(-4.59–-3.87)^a^ 32 months: -0.83(-1.14–-0.52)^a^	NA
					HOOS Symptoms			**Pre-op:** -4.01(-4.37–-3.66)^a^ 32 months: -1.0(-1.31–-0.68)^a^	
					HOOS ADL			**Pre-op:** -3.33(-3.67–-2.99)^a^ 32 months: -0.5(-0.81–-0.19)^a^	
					HOOS Sport			**Pre-op:** -3.49(-3.84–-3.15)^a^ 32 months: -0.94(-1.25–-0.63)^a^	
					HOOS QOL			**Pre-op:** -4.15(-4.50–-3.79)^a^ 32 months: -1.42(-1.73–-1.10)^a^	
Maldonado et al.^56^ (2019) Retrospective case series	*n =* 16 81% 24y(7) 24 kg/m^2^ (6)	12/16	Concomitant hip arthroscopy and PAO Surgical indications: LCEA <18°, no evidence of severe chondral damage evidence on dGEMRIC MRA	Reverse’ PAO for acetabular retroversion. Surgical Contraindications: Advanced OA, Tönnis grade >1, active infection, skeletally immature (age <12 yr), MRI findings of significant chondral damage and subchondral cysts, ‘older age groups’	iHOT	NA	Minimum 5 years post-op	NA	Not estimable^b^
Matheney et al.^97^ (2009) Retrospective case series	*n =* 109 70% 27y(9) NR	14/16	PAO for DDH	Non-DDH. <5 years outcome data	WOMAC	NA	9 years post-op	NA	Not estimable^b^
McClincy et al.^72^ (2019) Retrospective cohort study	*n =* 49 94% 27y(8) 24 kg/m^2^(5)	12/16	Patients undergoing PAO with pain and a LCEA of 18–25°	Surgical contraindications: Bilateral procedures, Dysplasia caused by surgical excision of a proximal femoral tumor, Tönnis grade 2 OA without remaining cartilage–correct into the weight-bearing zone.	HOOS Pain	NA	Pre-op and 2 years post-op	NA	**2 years:** 1.01(0.67–1.45)^a^
					HOOS Symptoms				**2 years:** 0.8(0.44–1.16)^a^
					HOOS ADL				**2 years:** 0.78(0.42–1.14)^a^
					HOOS Sport				**2 years:** 1.03(0.64–1.42)^a^
					HOOS QOL				**2 years:** 1.3(0.85–1.7) ^a^
Mechlenburg et al.^73^ (2018) Prospective cohort study	*n =* 41 83% 29y(9) 23 kg/m^2^(5)	13/16	LCEA ⩽24°, Tönnis OA grade 0 or 1, spherical femoral heads, painful hip, ⩾110° hip flexion and living <70 km away from the hospital	LCPD, previous PAO or other hip surgery on the affected leg, age <18 years	HAGOS	NA	Pre-op, 4 months and 12 months post-op	NA	Not estimable but favoured follow-up^b^
Mechlenburg et al.^55^ (2015) Prospective cohort study	26 NR NR NR	15/16	Scheduled for PAO for DDH	Metal implants, neurologic illnesses, LCPD, previous corrective paediatric hip surgery	HOOS	NA	Pre-op and 10 years post-op	NA	Not estimable^b^
Millis et al.^98^ (2009) Retrospective case series	*n =* 70 NR 44y(R40–51) NR	9/16	Symptomatic DDH, >40 years of age at time of surgery	Acetabular dysplasia secondary–Down’s syndrome, inflammatory arthritis, LCPD, neuromuscular diagnoses, or isolated acetabular retroversion	WOMAC	NA	Pre-op and mean 5 years post-op (R2–14)	NA	Not estimable^b^
Møse et al.^99^ (2019) Prospective cohort study	*n =* 99 92% 34y(R14–59) NR	12/16	Patients following PAO. Surgical indications: persisting hip pain, LCEA <25°, pelvic bone maturity, IR >15°, hip flexion <110^o^ and Tönnis OA grade 0 or 1	Multiple complaints from several joints, failure–show up at 2-year follow-up	WOMAC Pain	Dysplasia (LCEA <20 ^o^) vs Borderline Dysplasia (LCEA 20 ^o^ -24 ^o^)	Pre-op and 2 years post-op	**Pre-op:** 0.2(-0.23–0.63)^b^ 2 years: 0.0(-0.43–0.48)^b^	NA
Mortensen et al.^100^ (2018) Feasibility study	*n =* 16 75% 28y(R22–40) 24.3kg/m^2^	12/16	Patients with DDH on waiting list for PAO and living within 50 km of Aarhus University Hospital, able–transport themself–the study location, and age ⩾18 years	Tönnis OA score > 1; retroverted acetabulum, LCPD and epiphyseolysis; previous surgery for a herniated disc and spondyloses; lower limb joint preserving or arthroplasty of the hip, knee; neurological or rheumatological diseases affecting hip function; tenotomy of the iliopsoas tendon or z-plastic of the iliotibial band; BMI⩾ 40 kg/m^2^	HAGOS Pain	NA	Pre- and post strengthening programme	NA	9.5(0.9–18.1)^b^
					HAGOS Symptoms				12.1(2.9–21.2)^b^
					HAGOS ADL				Reported no significant change^b^
					HAGOS Sport				12.5(4.0–21.0)^b^
					HAGOS Participation				Reported no significant change^b^
					HAGOS QOL				7.5(1.7–13.3)^b^
Novais et al.^75^ (2018) Retrospective case-control study	*n =* 104 94% 25y(8) 24 kg/m^2^(4) / *n =* 52 94% 25y(7) 24 kg/m^2^(5)	13/16	Patients undergoing PAO. Surgical criteria: DDH; no previous hip surgery; no concurrent femoral osteotomy procedure / PAO residual or persistent pain after an ipsilateral hip arthroscopy and the diagnosis of DDH based on LCEA <25° or acetabular roof inclination of Tönnis >10°	Diagnosis different than DDH; previous open surgeries.	WOMAC Pain	PAO following arthroscopy	Pre-op and >1 year post-op	**Pre-op:** 0.35(0.01–0.68)^a^ 1 year: 0.56(0.22–0.9)^b^	NA
					WOMAC Stiffness			**Pre-op:** -0.77(-1.49–-0.05)^a^ 1 year: 0.38(0.05–0.72)^b^	
					WOMAC Function			**Pre-op:** 0.36(0.03–0.70)^a^ 1 year: 0.44(0.10–0.77)^b^	
Novais et al.^74^ (2013) Retrospective case series	*n =* 51 92% 27y(11) 24 kg/m^2^(4)	12/16	Symptomatic DDH with complete data; hip pain for at least 3 months; LCEA <16°, ACEA <20°, or both; and Tönnis Grade 0–2	Tönnis Grade >3; other significant hip condition; neuromuscular disease; incomplete data or medical records	WOMAC	NA	Pre-op, 1 year post-op and 2 years post-op	NA	Not estimable^b^
Novais et al.^82^ (2018) Retrospective case series	*n =* 33 100% 20y(6) 23 kg/m^2^(3)	12/16	Only female patients, >1-year follow-up post-op, participation in dance	Surgical contraindications: Tönnis OA grade 2	HOOS	NA	Pre-op and median 2.7 years post-op	NA	Not estimable but trend towards improvement at follow-up
Okoroafor et al.^76^ (2019) Retrospective case series	*n =* 58 72% 25y(R14–47) 24 kg/m^2^(R19–32)	13/16	PAO for symptomatic DDH, not improving after 3 months of activity modification, physical therapy, NSAIDS and intra-articular CSI; radiographic evidence of femoral head uncovering; LCEA <25° were indicated for surgery UCLA score of 7 preoperatively; ⩾5 years of follow-up data	Other significant hip condition; UCLA score <7; history of trauma; neuromuscular or connective tissue disorder’ previous surgery; Tönnis grade 2 or 3; LCPD; SCFE	WOMAC Pain	NA	Pre-op and 7 years post-op	NA	1.13(0.79–1.47)^b^
					WOMAC Stiffness				0.64(0.35–0.93)^b^
					WOMAC Function				0.84(0.54–1.15)^b^
Petrie et al.^77^ (2020) Retrospective case study	*n =* 359 77% 25y(R10–54) 25 kg/m^2^(R17–47)	11/16	Primary diagnosis of symptomatic acetabular dysplasia with minimum 2-year follow-up	Other significant hip conditions; neuromuscular disorders, and a history of prior ipsilateral pelvic osteotomy	HOOS	NA	Pre-op and mean 45 months post-op (R20–91)	NA	Not estimable but favoured follow-up^b^
Ramírez-Núñez et al.^78^ (2020) Retrospective study	*n =* 118 78% 32y(10) NR	12/16	Patients with persistent mechanical hip pain, DDH, congruent joint surfaces, joint space greater than 3 mm, hip flexion greater than110^0^ and IR < 15^0^	Missing radiological or functional information	NAHS	NA	Pre-op and mean 7.7 years post-op	NA	2.86(2.44–3.26)^b^
Ricciardi et al.^83^ (2017) Retrospective case-control study	*n =* 27/*n =* 50 100%/88% 25y(R15–43)/23y(R12–41) 22(R18–36) kg/m^2^ / 23 kg/m^2^ (R17–30)	15/18	Patients undergoing PAO. >6/12 postoperative from final hip surgery. Surgical indications: DDH (LCEA <25), pain that has failed ⩾6 weeks of conservative management including physical therapy and NSAIDs, joint congruency, and Tönnis grade 0-1	Patients undergoing bilateral PAO with unilateral borderline dysplasia	iHOT-33	Mild DDH / Severe DDH	Pre-op, 6 months and 1 year post-op	NA	Not estimable but favoured follow-up^b^
Ricciardi et al.^101^ (2017) Retrospective case-control study	*n =* 22 91% 27y(R18–41) 22 kg/m^2^(3)	16/18	PAO for symptomatic DDH, >6 months from last hip surgery, with pre-op hip-specific functional outcome, minimum 1-year clinical follow-up from their first PAO	NR	iHOT-33	PAO following previous arthroscopy	Pre-op, 6 months and 1 year post-op	**Pre-op:** 0.17(-0.31–0.65)^b^ 6 months: 1.08(0.43–1.74)^b^ 1 year: 0.83(0.22–1.44)^b^	NA
Ricciardi et al.^102^ (2016) Retrospective case-control study	*n =* 52/*n =* 21 89%/100% 23y(R12–43)/27y(R12–41) 23 kg/m^2^(3)/ 22 kg/m^2^(3)	14/18	symptomatic DDH undergoing PAO, >6 months post-op from last hip surgery, with pre- and post-op outcome scores	Combined hip arthroscopy with labral debridement alone, and patients with a scope/PAO on 1 hip with a contralateral PAO alone	iHOT-33	PAO / Scope + PAO	Pre-op, 6 months and 1 year post-op	**Pre-op:** 0.46(-0.05–0.97)^b^ 6-months: 0.10(-0.51–0.71)^b^ 1 year: -0.41(-1.09–0.27)^b^	NA
Ross et al.^54^ (2014) Retrospective cohort study	*n =* 30 77%/87% 24y/27y 25 kg/m^2^/24 kg/m^2^(R18–35)	7/18	Symptoms that were refractory–non-operative treatment, a physical examination and radiographic findings consistent with acetabular dysplasia and a complete data set	NR	WOMAC	PAO/PAO post-scope	Pre-op	Not estimable but favoured PAO group^b^	NA
Scott et al.^85^ (2020) Cross-sectional cohort study	*n =* 24/21 100%/91% 24y(9)/25y(6) 24 kg/m^2^(4)/24 kg/m^2^(3)	13/18	Aged 15–39 years with DDH (LCEA <25°) scheduled for treatment with PAO	PAO exclusively for acetabular retroversion, neuromuscular condition, history of Perthes disease, Tönnis grade >1, or previous open hip surgery were excluded	HOOS Pain	Healthy Participants	Pre-op	-4.84(-6.09–-3.6)^a^	NA
Swarup et al.^58^ (2018) Retrospective cohort study	*n =* 33 97% 17y(2) 21.2 kg/m^2^(4)	10/16	Primary PAO or PAO following hip arthroscopy with a minimum of 1 year follow-up. Surgical indications: aged ⩽21, a LCEA ⩾18 and ⩽25. Patients undergoing bilateral PAO were included in this study if their outcomes were at least 12 months from the second sided surgery.	Patients that had missing baseline or 1-year follow-up data, patients that underwent anteverting PAO	iHOT-33	NA	Pre-op and 1-year post-op	NA	3.32(2.45–4.19)^b^
Selberg et al.^103^ (2020) Retrospective study	*n =* 314 85% 24y(9) NR	12/18	Patients with symptoms > 3 months preoperatively and DDH with a LCEA <25°, minimum of 12 months postoperatively	Reverse PAO or PAO due–skeletal chondrodysplasia	HOOS	Non-union following PAO	Pre-op and 12+ months post-op	Not estimable^b^	NA
Thanacharoenpanich et al.^104^ (2018) Retrospective case-control study	*n =* 47/*n =* 60 87%/92% 25y(10)/31y(9) 25 kg/m^2^(4)/25 kg/m^2^(4)	14/18	Skeletally mature DDH patients (LCEA<20° and/or ACEA <20°) with a full thickness labral tear on preoperative MRA, ⩾1 year of follow-up after surgery. Surgical indications: ⩾3 months of hip and/or groin pain aggravated by activity, despite non-operative management	Any syndromic form of DDH and those who had incomplete data	HOOS Pain	PAO / PAO+A (Arthrotomy or Arthroscopy)	Pre-op and mean 2.1 years post-op (R1–3)	**Pre-op:** 0.45(0.06–0.84)^b^ 2 years: 0.13(-0.25–0.51)^b^	NA
					HOOS Symptoms			**Pre-op:** 0.68(0.29–1.07)^b^ 2 years: 0.18(-0.21–0.56)^b^	
					HOOS ADL			**Pre-op:** 0.42(0.03–0.8)^b^ 2 years: 0.22(-0.17–0.6)^b^	
					HOOS Sport			**Pre-op:** 0.61(0.22–1.0)^b^ 2 years: 0.3(-0.08–0.69)^b^	
					HOOS QOL			**Pre-op:** 0.60(0.21–0.99)^b^ 2-years: 0.37(-0.01–0.76)^b^	
Troelsen et al.^105^ (2009) Retrospective case series	*n =* 96 78% 30y 24 kg/m^2^(R15–37)	11/16	Surgical indications: symptomatic acetabular dysplasia defined by persistent pain, a CEA of <25°, a congruent hip joint, hip flexion of >110°, and IR of >15°	Unavailable for follow-up	WOMAC	NA	Mean 6.8 years (R5–9)	NA	Not estimable^b^
Wasko et al.^31^ (2019) Prospective observational cohort study	*n =* 294 84% 21y 23 kg/m^2^	8/16	Aged 18–40 years and undergone PAO for acetabular dysplasia and returned for 1 year follow-up	PAO for diagnoses other than DDH or if they presented with other lower-limb injuries at any time point	HOOS Pain	NA	Pre-op, 1 year and 2 years post-op	NA	**1-year:** 1.5(1.33–1.67)^a^ **2-years:** 1.57(1.4–1.74)^a^
					HOOS Symptoms				**1-year:** 1.21(1.06–1.36)^a^ **2-years:** 1.22(1.07–1.37)^a^
					HOOS ADL				**1-year:** 1.38(1.22–1.54)^a^ **2-years:** 1.18(1.03–1.32)^a^
					HOOS Sport				**1-year:** 1.41(1.25–1.57)^a^ **2-years:** 1.54(1.37–1.71)^a^
					HOOS QOL				**1-year:** 1.38(1.22–1.54)^a^ **2-years:** 1.45(1.28–1.61)^a^
Wells et al.^79^ (2018) Retrospective case series	*n =* 154 86% 26yR10–60) 24 kg/m^2^(R17–34)	13/16	PAO for symptomatic DDH	Neuromuscular or connective-tissue disorder, prior trauma, additional diagnoses other than DDH	WOMAC Pain	Preserved vs symptomatic hips	Pre-op and ⩾6 months post-op	3.06(2.49–3.62)^b^	NA
Wells et al.^106^ (2019) Retrospective case series	*n =* 129 86% 26y 24 kg/m^2^(R17–34)	12/16	PAO for symptomatic DDH. Surgical criteria: symptomatic DDH, radiographic evidence of femoral head uncovering, and a LCEA of <25°	Neuromuscular or connective-tissue disorder, prior trauma, additional diagnoses other than DDH	WOMAC	NA	Mean 10 years post-op (R1.7–20.5)	NA	Not estimable^b^
Wells et al.^2^ (2017) Retrospective cohort study	*n =* 64/*n =* 31 83%/93% 25y(9)/27y(8) 26 kg/m^2^(5)/28 kg/m^2^(7)	13/16	PAO patients. Surgical criteria: Patients with hip pain and radiographic evidence of femoral head uncovering and a LCEA <20° as well as closed triradiate cartilage	Hip trauma, neuromuscular or connective tissue disorder. Surgical contraindications: OA without remaining cartilage–correct into the weightbearing zone or prior hip trauma.	HOOS	PAO Asymptomatic/ PAO Symptomatic	Pre-op and mean 18 years (R14–22) post-op	Not estimable^b^	NA
Wyles et al.^50^ (2018) Retrospective cohort study	*n =* 71 87% 27y(7) 25 kg/m^2^(5)	10/18	Surgical criteria: closed triradiate cartilage and symptomatic DDH	Isolated acetabular retroversion, neurogenic dysplasia, LCPD or SCFE	HOOS Pain	PAO Arthrotomy / PAO Arthroscopy	Pre-op and post-op (unspecific)	**Pre-op:** -0.02(-0.49–0.45)^b^ Follow-up: -0.57(-1.05–-0.09)^b^	NA
					HOOS Symptoms			**Pre-op:** -0.11(-0.57–0.36)^b^ Follow-up: -0.53(-1.01–-0.05)^b^	
					HOOS ADL			**Pre-op:** -0.02(-0.49–0.45)^b^ Follow-up: -0.37(-0.84–0.10)^b^	
					HOOS Sport			**Pre-op:** -0.17 (-0.64–0.30)^b^ Follow-up: -0.39(-0.86–0.08)^b^	
					HOOS QOL			**Pre-op:** -0.03(-0.49–0.44)^b^ Follow-up: -0.69(-1.18– -0.21)^b^	
Ziebarth et al.^80^ (2011) Retrospective cohort study	*n =* 38 0% 24y(10) NR	14/16	PAO for DDH. Surgical indications: pain, femoral head uncovering on the AP radiograph with LCEA <20°	Surgical contraindications: OA, open triradiate cartilage, prior surgery	WOMAC Pain	NA	Pre-op and mean 3 years post-op (R1–7.6)	NA	1.36(0.86–1.87)^b^
					WOMAC Stiffness				0.53(0.14–0.92)^b^
					WOMAC Function				0.04(-0.32–0.41)^b^
Ziran et al.^107^ (2019) Retrospective cohort study	*n =* 258 83% 32y(10) NR	12/18	Surgical indications: >6 months of pain in the involved hip, adequate range of motion, Tönnis grade 1-2 or less with some exceptions, LCEA <20°, and closure of the triradiate cartilage	NR	HOOS	NA	Pre-op and mean 11.2 years (R0.25–28 years)	NA	Not estimableb

ACEA, anterior centre-edge angle; ADL, activities of daily living; AP, anteroposterior; CEA, centre-edge angle; CSI, corticosteroid injections; DDH, developmental dysplasia of the hip; FAI, femoroacetabular impingement; HAGOS, Copenhagen Hip and Groin Outcome Score; HOOS, Hip disability and Osteoarthritis Outcome Score; iHOT, International Hip Outcome Tool; IR, internal rotation; LCEA, lateral centre-edge angle; LCPE, Legg-Calvé-Perthes disease; MRI, magnetic resonance imaging; MRA, magnetic resonance arthrography; NA, not applicable; NAHS, Non-arthritic Hip Score; NR, not reported; NSAIDs, nonsteroidal anti-inflammatory drugs; PROM, patient-reported outcome measure; QOL, quality of life; ROM, range of motion; SCFE, slipped capital femoral epiphysis; UCLA, University of California Los Angeles; WOMAC, Western Ontario and McMaster Universities Osteoarthritis Index.

a Result also included in meta-analyses.

b Result not included in meta-analyses for one of the following reasons: single study for a particular outcome or timepoint, same cohort as another study, incomplete data provided.

### PAO versus healthy controls

**Figure 2.**
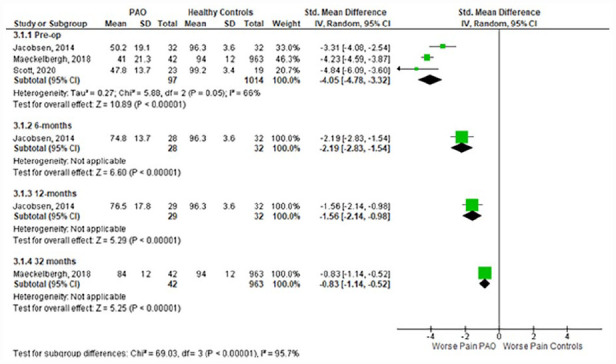
Forest plot comparing **Pain** subscale scores in those undergoing PAO and healthy controls. Interpretation of findings: - 3.1.1 (pre-op) SMD = −4.05: large effect size (>0.8) showing worse Pain scores in PAO patients versus healthy controls preoperatively. - 3.1.2 (6 months) SMD = −2.19: large effect size (>0.8) showing worse Pain scores in PAO patients versus healthy controls at 6 months postoperatively. - 3.1.3 (12 months) SMD = −1.56: large effect size (>0.8) showing worse Pain scores in PAO patients versus healthy controls at 12 months postoperatively. - 3.1.4 (32 months) SMD = −0.83: large effect size (>0.8) showing worse Pain scores in PAO patients versus healthy controls at 32 months postoperatively. CI, confidence interval; IV, Random, random effects model; Std, standardised; SD, standard deviation; SMD, standardised mean difference; PAO, periacetabular osteotomy.

**Figure 3.**
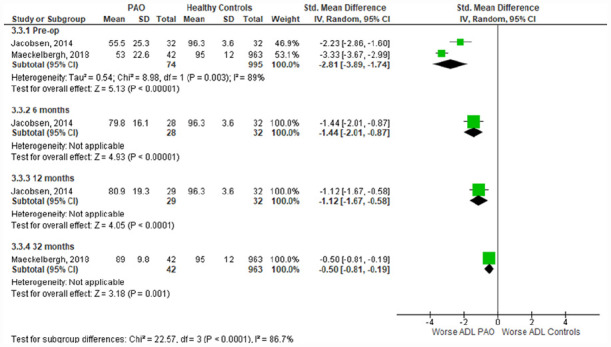
Forest plot comparing **Activities of Daily Living (ADL)** subscale scores in those undergoing PAO and healthy controls. Interpretation of findings: • 3.3.1 (pre-op) SMD = −2.81: large effect size (>0.8) showing worse ADL scores in PAO patients versus healthy controls preoperatively. • 3.3.2 (6 months) SMD = −1.44: large effect size (>0.8) showing worse ADL scores in PAO patients versus healthy controls at 6 months postoperatively. • 3.3.3 (12 months) SMD = −1.12: large effect size (>0.8) showing worse ADL scores in PAO patients versus healthy controls at 12 months postoperatively. • 3.3.4 (32 months) SMD = −0.5: moderate effect size (0.5) showing worse ADL scores in PAO patients versus healthy controls at 32 months postoperatively. CI, confidence interval; IV, Random, random effects model; Std, standardised; SD, standard deviation; SMD, standardised mean difference; PAO, periacetabular osteotomy.

**Figure 4.**
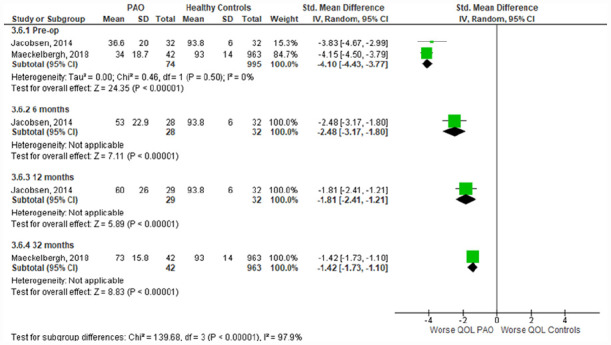
Forest plot comparing **Quality of Life** subscale scores in those undergoing PAO and healthy controls. Interpretation of findings: • 3.6.1 (pre-op) SMD = −4.1: large effect size (>0.8) showing worse QOL scores in PAO patients versus healthy controls preoperatively. • 3.6.2 (6 months) SMD = −2.48: large effect size (>0.8) showing worse QOL scores in PAO patients versus healthy controls at 6 months postoperatively. • 3.6.3 (12 months) SMD = −1.81: large effect size (>0.8) showing worse QOL scores in PAO patients versus healthy controls at 12 months postoperatively. • 3.6.4 (32 months) SMD = −1.42: large effect size (>0.8) showing worse QOL scores in PAO patients versus healthy controls at 32 months postoperatively. CI, confidence interval; IV, Random, random effects model; Std, standardised; SD, standard deviation; SMD, standardised mean difference; QOL, quality of life; PAO, periacetabular osteotomy.

2 studies compared outcomes pre-operatively and postoperatively, between PAO patients and healthy controls, 1 high-quality prospective cohort study, and 1 high quality cross-sectional study.^19,84^ An additional high-quality cross-sectional study also compared the pain subscale only, between patients with DDH and healthy controls, preoperatively.^85^ Meta-analysis of the preoperative time-point showed significantly worse pain (SMD [95% CI]: −4.05; −4.78 to −3.32) (Figure 2), activities of daily living (−2.81; −3.89 to −1.74) (Figure 3), QOL (−4.10; −4.43 to −3.77) (Figure 4), symptoms (−3.84; −4.36 to −3.29) (Supplemental Appendix 3), and sport & recreation (−3.47; −3.79 to −3.16) (Supplemental Appendix 4), for those undergoing PAO versus controls. Despite the large magnitude of effect found, the GRADE level of certainty of these pre-operative difference between PAO patients and healthy controls is low. This is due to inconsistency in data and risk of bias.^45^

Data were unable to be pooled for the postoperative time points as the studies used different follow-up time periods. Jacobsen et al.^19^ reported follow-up data at 6 months and 12 months postoperatively, and Maeckelbergh et al.^84^ reported 32 months postoperative data. Across all subgroups the PAO group had significantly worse outcomes than the healthy controls, at every time point. The magnitude of difference between healthy controls and those undergoing PAO has significantly reduced with time following PAO (*p* < 0.001).

### Change from pre-op to post-PAO

9 studies measured change in pain in their respective cohorts following PAO (Supplemental Appendix 5). Meta-analysis of 3 studies reported an improvement at the 1-year time-point (SPD 1.35; 95% CI, 1.02–1.67; I^2^ = 80%). This included a prospective observational study,^31^ a high-quality retrospective case series study,^85^ and a high-quality prospective case series study.^36^

A similar result was found at the 2-year timepoint (1.35; 1.16–1.54; I^2^ = 64%) with meta-analysis of 4 studies. A prospective observational study,^31^ and 3 respective cohort studies,^33^ 2 of which were high-quality.^30,72^ Other included studies also reported improvement in pain at different timepoints, but meta-analysis was not possible at these timepoints as only single studies assessed the timepoint as shown in Supplemental Appendix 5.

Changes in activities of daily living (ADL) following PAO was measured in 5 studies (Supplemental Appendix 6). Meta-analysis of a prospective observational cohort study and a high-quality prospective case series showed improvement at the 1-year time-point (1.22; 1.09–1.35; I^2^ = 0%).^31,36^ This was also shown at the 2-year time-point (1.06; 0.90–1.22; I^2^ = 53%) with meta-analysis of 3 studies, 2 high-quality retrospective cohort studies and a prospective observational study. A single study also showed improvement at the 1.5 year timepoint (1.63; 1.23–2.02).

Improvements following surgery were also observed for QOL at 1-year (1.36; 1.22–1.5; I^2^ = 0%) and 2-year (1.3; 1.1–1.5; I^2^ = 65%) time-points (Supplemental Appendix 7), sport and recreation at 1-year (1.29; 1.01–1.57; I^2^ = 68%) and 2-year (1.24; 0.92–1.57; I^2^ = 87%) time-points (Supplemental Appendix 8), and symptoms at 1-year (1.16; 1.01–1.32; I^2^ = 16%) and 2-year (1.02; 0.79–1.25; I^2^ = 77%) time-points (Supplemental Appendix 9).

The improvements found across all subgroups from pre- to post- PAO surgery provide low level certainty that pain, ADL, QOL, sport and recreation, and symptoms improve following surgery. Despite the large magnitude of effect found, risk of bias of studies and inconsistency in data means the GRADE certainty rating remains at low.^45^

### Primary PAO versus PAO following arthroscopy

3 high-quality studies compared outcomes between those having a PAO as their first hip surgery, and those having a PAO following a previous arthroscopy.^81–83^ We were able to perform meta-analyses of 2 studies.^81,82^ The observational methodology of the studies means these results provide low level certainty that there was no significant difference preoperatively between both groups in pain (SMD 0.15; 95% CI, −0.38 to 0.68) (Supplemental Appendix 10), stiffness (−0.29; −1.10 to 0.52) (Supplemental Appendix 11) and function (−0.03; 0.90 to 0.84) (Supplemental Appendix 12).

1 study provided limited evidence showing despite similar baseline values (0.17; −0.31 to 0.65), those with previous arthroscopy had significantly worse outcomes at 6 months (1.08; 0.43 to 1.74) and 1 year (0.83; 0.22 to 1.44) post PAO compared to those who had not had previous arthroscopy, as measured by the iHOT-33.^83^

### Mild versus severe dysplasia

2 high-quality studies dichotomised their groups by their lateral centre-edge angle (LCEA) measurement.^99,101^ We were unable to pool the data from these studies because of differences in outcome measures used, and differences in the LCEA used to define both groups. Ricciardi et al.^83^ compared those with a LCEA of 18–25° to those with LCEA of ⩽17° using the iHOT-33 at preoperative (−0.06; −0.57 to 0.45), 6-month (0.10 (−0.47 to 0.66) and 1-year time-points 0.05 (−0.64 to 0.73).^101^ Møse et al.^99^ compared those with a LCEA of 20–25° to those with a LCEA of <20° using the WOMAC Pain subscale preoperatively (0.20; −0.23 to 0.63) and at 2 years postoperative (0.00; −0.43 to 0.48).

This limited evidence shows no significant difference between groups with DDH pre-operatively or following surgery when dichotomised using LCEA.

## Discussion

Our systematic review aimed to evaluate pain, function, and QOL in adults with DDH undergoing PAO, as assessed by PROMs. We found low level evidence that those with DDH undergoing PAO had significantly worse PROMs (pain, symptoms, activities of daily living, sport & recreation, and quality of life) preoperatively compared with healthy participants. Patients do improve following PAO surgery, and these improvements appear to be maintained for the 7 years of data we have available. Despite these improvements, post-operatively patients do not return to the same level of pain, function, and QOL as healthy participants for the 3-year period following surgery.

We dichotomised results for patients with DDH based on their LCEA, into mild versus severe dysplasia. There is growing recognition that the diagnosis of DDH is more complex than just examining the LCEA, and probably involves multiple variables in the pattern of dysplastic morphology.^3^ The Ottawa classification system, as an example, identifies a proportion of dysplastic patients who have no lateral acetabular deficiency.^16^ There is also a greater recognition that variations in hip morphology are common in those who do not have symptoms,^108,109^ implying that while morphology is a factor potentially influencing the severity of a patient’s symptoms, it may not be the primary driver of pain.

Surgical complications are known to affect pain and activity in these patients but was not evaluated in this systematic review.^26,27^ Though the PAO is considered a safe procedure with low levels of complications,^106,110^ a recent study reported bony non-union as the most common major surgical complication at 12%, with 26% of these patients being symptomatic requiring open reduction and internal fixation.^110^

There are a number of orthobiologic products utilised in other surgeries that have been shown to enhance bone grafts and provide higher rates of fusion in spinal orthopaedic surgery.^111^ However, it is unknown whether these are effective in PAO surgery or improve outcomes such as pain or QOL. There has also been no synthesis of the evidence in relation to PAO surgery. Understanding complications that potentially affect long-term pain and activity and possible solutions for such complications warrants further investigation.

We only investigated differences in patient-reported outcomes, between patients with DDH undergoing PAO and healthy participants in this systematic review. However, similar deficits have been shown in this cohort in individual studies investigating physical impairments.^5,15,26,31,32,34,60^ A synthesis of the evidence relating to physical impairments would provide greater understanding of how these patients present physically. Understanding physical impairments may also help inform pre- and postoperative rehabilitation by allowing clinicians to target these impairments in rehabilitation programs. These young adults may wish, and should be encouraged, to return to sport and physical activity.^10^ While this was not investigated in our review, future studies should explore this important domain.

This review contains several limitations that should be acknowledged. Firstly, there were no randomised controlled trials, and a large proportion of retrospective studies, which have implications for introducing selection, performance and detection bias. Included studies demonstrated considerable variability in the risk of bias, outcomes reported, and post-operative assessment timepoints, which limited opportunities for meta-analysis. Included studies had poor transparency in describing characteristics of patients lost to follow-up, and a lack of validity and reliability for main outcome measures. The above factors rendered it impossible to obtain findings with ‘high’ level evidence and certainty ratings.^43^ Longitudinal studies are critical to investigate potential causality and better understand the relationships between pain, function and QOL in patients with DDH undergoing PAO.

Adults with DDH undergoing PAO have more pain and worse function and QOL scores compared to healthy participants. Patients do improve following PAO surgery, and maintain this improvement, but they do not to the same level as their healthy participants. Our findings are important to patients and clinicians when considering PAO surgery, to appropriately manage expectations of recovery, thus enhancing the shared decision-making process, weighing up benefits of surgery against risks.

## Supplemental Material

sj-pdf-1-hpi-10.1177_11207000231179610 – Supplemental material for Pain, function and quality of life are impaired in adults undergoing periacetabular osteotomy (PAO) for hip dysplasia: a systematic review and meta-analysis Click here for additional data file. Supplemental material, sj-pdf-1-hpi-10.1177_11207000231179610 for Pain, function and quality of life are impaired in adults undergoing periacetabular osteotomy (PAO) for hip dysplasia: a systematic review and meta-analysis by Michael JM O’Brien, Adam I Semciw, Inger Mechlenburg, Lisa CU Tønning, Chris JW Stewart and Joanne L Kemp in HIP International
