# Supplementary material for: Pain, function and quality of life are impaired in adults undergoing periacetabular osteotomy (PAO) for hip dysplasia: a systematic review and meta-analysis
Source: Hip Int. 2023 Jun 12;34(1):96–114. doi: 10.1177/11207000231179610 (PMC10787396; doi:10.1177/11207000231179610)
Supplement: sj-pdf-1-hpi-10.1177_11207000231179610 – Supplemental material for Pain, function and quality of life are impaired in adults undergoing periacetabular osteotomy (PAO) for hip dysplasia: a systematic review and meta-analysis [file sj-pdf-1-hpi-10.1177_11207000231179610.pdf]

Pain, Function and Quality of Life are impaired in young active adults undergoing Periacetabular Osteotomy (PAO) for Developmental Dysplasia of the Hip (DDH): A Systematic Review and Meta-Analysis

Supplementary Appendices

Supplementary appendix 1:

The search strategy for MEDLINE was as follows:
("Joint Instability"[Mesh] OR instability[tiab] OR unstable[tiab] OR lax[tiab] OR laxity[tiab] OR subluxation[tiab] OR dysplasia[tiab] OR dysplastic[tiab] OR anterversion[tiab] OR retroversion[tiab]) AND (Periacetabular [Mesh] OR periacetabular [tiab] OR Osteotomy[Mesh] OR osteotomy [tiab]) AND (Hip[Mesh] OR Hip[tiab] OR hips[tiab] OR "Hip Joint"[Mesh] OR "coxofemoral joint"[tiab]) OR Pelvis [Mesh] OR Pelvis [tiab] OR Pelvic [Mesh] OR Pelvic [tiab])

Supplementary appendix 2:

Quality Appraisal results table

Author, Year	Criteria																		Total	Percentage
	1	2	3	4	5	6	7	8	9	10	11	12	13	14	15	16	17	18		
Mechlenburg, 2015																			15 / 16	93.8
Ricciardi, 2017b																			16 / 18	88.9
Larsen, 2020																			14/16	87.5
Ziebarth, 2011																			14 / 16	87.5
Matheney, 2009																			14 / 16	87.5
Ricciardi, 2017a																			15 / 18	83.3
Wells, 2018																			13 / 16	81.3
Wells, 2017																			13 / 16	81.3
Mechlenburg, 2018																			13 / 16	81.3

Jacobsen, 2019	13 / 16	81.3
Hartig-Andreasen, 2012	13 / 16	81.3
Okoroafor, 2019	13/16	81.3
Grammatopoulos, 2016	13 / 16	81.3
Dahl, 2014	13 / 16	81.3
Thanacharoenpanich, 2018	14 / 18	77.8
Ricciardi, 2016	14 / 18	77.8
Jacobsen, 2014	14 / 18	77.8
Hartig-Andreasen, 2015	14 / 18	77.8
Wells, 2019	12 / 16	75
Mortensen, 2018	12/16	75.0
Mose, 2019	12 / 16	75
McClincy, 2019	12 / 16	75
Novais, 2018b	12 / 16	75
Novais, 2013	12 / 16	75
Goronzy, 2020	12 / 16	75
Maldonado, 2019	12 / 16	75
Khan, 2017	12 / 16	75
Ramirez-Nunez, 2020	12/16	75.0
Clohisy, 2017	12 / 16	75
Beaule, 2015	12 / 16	75
Scott, 2020	13/18	72.2
Novais, 2018a	13 / 18	72.2
Maeckelbergh, 2018	13 / 18	72
Edelstein, 2020	11/16	68.8
Troelsen, 2009	11 / 16	68.8
Heyworth, 2016	11 / 16	68.8
Petrie, 2020	11/16	68.8

Duncan, 2015																			11 / 16	68.8
Grammatopoulos, 2018																			11 / 18	68.8
Brusalis, 2020																			12/18	66.7
Ziran, 2019																			12 / 18	66.7
Goronzy, 2017																			12 / 18	66.7
Selberg, 2020																			12/18	66.7
Biedermann, 2008																			12 / 18	66.7
Hingsammer, 2015																			10 / 16	62.5
Karam, 2011																			10 / 16	62.5
Cates, 2019																			10 / 16	62.5
Bogunovic, 2014																			10 / 16	62.5
Li, 2020																			10/16	62.5
Boje, 2019																			10 / 16	62.5
Swarup, 2020																			10/16	62.5
Hsieh, 2009																			11 / 18	61.1
Kain, 2011																			11 / 18	61.1
Belzile, 2016																			11/ 18	61.1
Millis, 2009																			9 / 16	56.3
Kralj, 2005																			9 / 16	56.3
Wyles, 2018																			10 /18	55.6
Wasko, 2019																			8 / 16	50
Garbuz, 2008																			9 / 18	50
Domb, 2015																			8 / 16	50
Davidson, 2011																			8 / 16	50
Ross, 2014																			7 / 18	38.9
**Total**	**61**	**60**	**52**	**48**	**53**	**59**	**52**	**34**	**8**	**57**	**57**	**30**	**25**	**59**	**16**	**31**	**16**	**14**		

Dark grey = low bias risk, White= high bias risk, Light grey = not applicable

Supplementary appendix 3:

Study or Subgroup	PAO Mean	PAO SD	PAO Total	Healthy Controls Mean	Healthy Controls SD	Healthy Controls Total	Weight	Std. Mean Difference IV, Random, 95% CI
**3.2.1 Pre-op**								
Jacobsen, 2014	49.3	17	32	92.8	5.1	32	31.7%	-3.42 [-4.21, -2.64]
Maeckelbergh, 2018	39	17.2	42	92	13	963	68.3%	-4.01 [-4.37, -3.66]
**Subtotal (95% CI)**			**74**			**995**	**100.0%**	**-3.83 [-4.36, -3.29]**
Heterogeneity: Tau² = 0.08; Chi² = 1.80, df = 1 (P = 0.18); I² = 44%								
Test for overall effect: Z = 13.97 (P < 0.00001)								
**3.2.2 6 months**								
Jacobsen, 2014	66.96	17.6	28	92.8	5.1	32	100.0%	-2.03 [-2.66, -1.40]
**Subtotal (95% CI)**			**28**			**32**	**100.0%**	**-2.03 [-2.66, -1.40]**
Heterogeneity: Not applicable								
Test for overall effect: Z = 6.30 (P < 0.00001)								
**3.2.3 12 months**								
Jacobsen, 2014	69.82	18.5	29	92.8	5.1	32	100.0%	-1.71 [-2.30, -1.12]
**Subtotal (95% CI)**			**29**			**32**	**100.0%**	**-1.71 [-2.30, -1.12]**
Heterogeneity: Not applicable								
Test for overall effect: Z = 5.66 (P < 0.00001)								
**3.2.4 32 months**								
Maeckelbergh, 2018	79	13.8	42	92	13	963	100.0%	-1.00 [-1.31, -0.68]
**Subtotal (95% CI)**			**42**			**963**	**100.0%**	**-1.00 [-1.31, -0.68]**
Heterogeneity: Not applicable								
Test for overall effect: Z = 6.26 (P < 0.00001)								

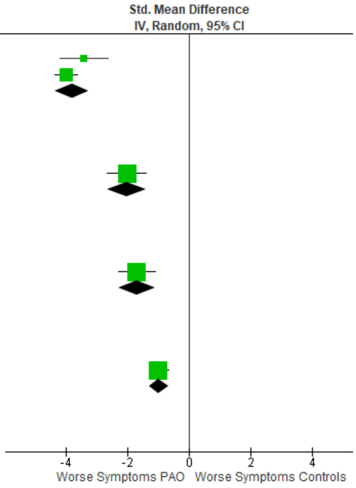

Test for subgroup differences: Chi² = 80.65, df = 3 (P < 0.00001), I² = 96.3%

Forest plot comparing **Symptoms** subscale scores in those undergoing PAO and healthy controls. Abbreviations: CI, confidence interval; IV, Random, random effects model; Std, standardised; SD, standard deviation; PAO, periacetabular osteotomy

Supplementary appendix 4:

Study or Subgroup	PAO Mean	PAO SD	PAO Total	Healthy Controls Mean	Healthy Controls SD	Healthy Controls Total	Weight	Std. Mean Difference IV, Random, 95% CI
**3.4.1 Preo-op**								
Jacobsen, 2014	39.2	23.2	32	96	3.9	32	16.4%	-3.37 [-4.15, -2.60]
Maeckelbergh, 2018	28	18.7	42	91	18	963	83.6%	-3.49 [-3.84, -3.15]
**Subtotal (95% CI)**			**74**			**995**	**100.0%**	**-3.47 [-3.79, -3.16]**
Heterogeneity: Tau² = 0.00; Chi² = 0.07, df = 1 (P = 0.78); I² = 0%								
Test for overall effect: Z = 21.60 (P < 0.00001)								
**3.4.2 6 months**								
Jacobsen, 2014	63.2	20.2	28	96	3.9	32	100.0%	-2.30 [-2.96, -1.64]
**Subtotal (95% CI)**			**28**			**32**	**100.0%**	**-2.30 [-2.96, -1.64]**
Heterogeneity: Not applicable								
Test for overall effect: Z = 6.81 (P < 0.00001)								
**3.4.3 12 months**								
Jacobsen, 2014	64.4	25.4	29	96	3.9	32	100.0%	-1.76 [-2.36, -1.16]
**Subtotal (95% CI)**			**29**			**32**	**100.0%**	**-1.76 [-2.36, -1.16]**
Heterogeneity: Not applicable								
Test for overall effect: Z = 5.78 (P < 0.00001)								
**3.4.4 32 months**								
Maeckelbergh, 2018	74	18.7	42	91	18	963	100.0%	-0.94 [-1.25, -0.63]
**Subtotal (95% CI)**			**42**			**963**	**100.0%**	**-0.94 [-1.25, -0.63]**
Heterogeneity: Not applicable								
Test for overall effect: Z = 5.92 (P < 0.00001)								

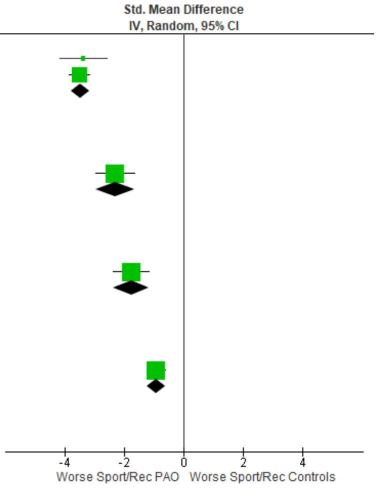

Test for subgroup differences: Chi² = 127.14, df = 3 (P < 0.00001), I² = 97.6%

Forest plot comparing **Sport & Recreation** subscale scores in those undergoing PAO and healthy controls.
Abbreviations: CI, confidence interval; IV, Random, random effects model; Std, standardised; SD, standard deviation; PAO, periacetabular osteotomy

Supplementary appendix 5:

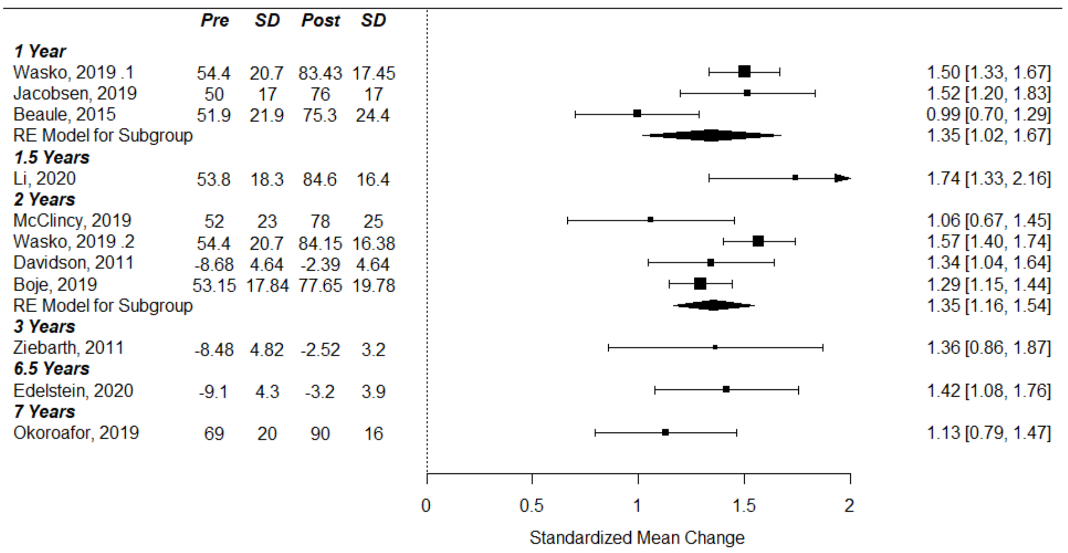

Forest plot measuring change in **Pain** subscale score following periacetabular osteotomy.
Abbreviations: RE, random effects model; SD, standard deviation

Supplementary appendix 6:

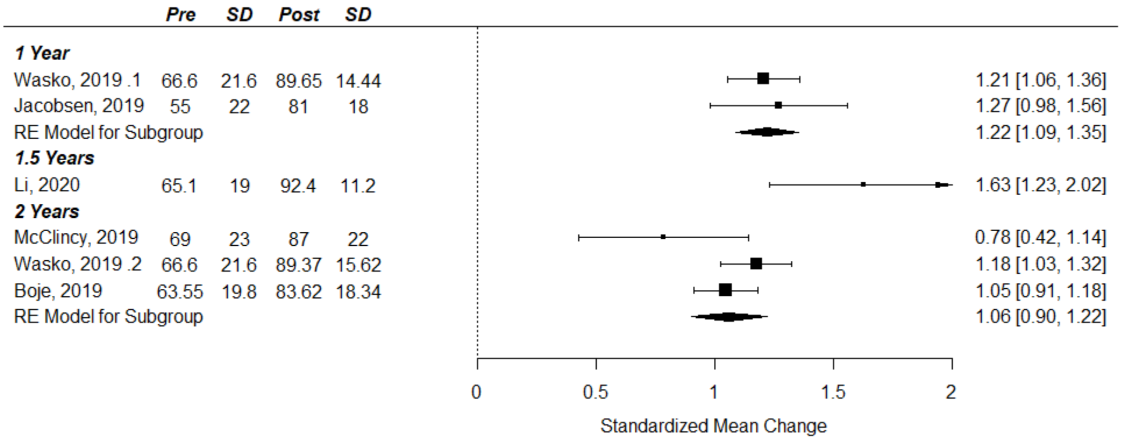

Forest plot measuring change in **Activities of Daily Living** subscale score following periacetabular osteotomy
Abbreviations: RE, random effects model; SD, standard deviation

Supplementary appendix 7:

	Pre	SD	Post	SD	
1 Year					
Wasko, 2019 .1	32.1	22.4	65.67	25.79	1.38 [1.22, 1.54]
Jacobsen, 2019	29	14	57	25	1.28 [0.99, 1.57]
RE Model for Subgroup					1.36 [1.22, 1.50]
1.5 Years					
Li, 2020	30.8	17.2	66.2	26.3	1.51 [1.13, 1.89]
2 Years					
McClincy, 2019	32	24	66	28	1.27 [0.85, 1.69]
Wasko, 2019 .2	32.1	22.4	65.82	24.06	1.45 [1.28, 1.61]
Boje, 2019	33.35	16.21	58.93	24.75	1.17 [1.03, 1.31]
RE Model for Subgroup					1.30 [1.10, 1.50]

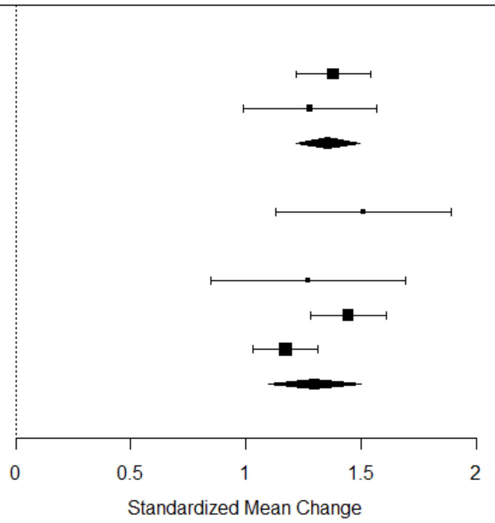

Forest plot measuring change in **Quality of Life** subscale score following periacetabular osteotomy
Abbreviations: RE, random effects model; SD, standard deviation

Supplementary appendix 8:

	Pre	SD	Post	SD	
1 Year					
Wasko, 2019 .1	40.9	25.3	75.41	23.3	1.41 [1.25, 1.57]
Jacobsen, 2019	39	21	64	23	1.12 [0.85, 1.40]
RE Model for Subgroup					1.29 [1.01, 1.57]
1.5 Years					
Li, 2020	40.8	19.6	77.7	19.8	1.85 [1.41, 2.28]
2 Years					
McClincy, 2019	47	29	76	26	1.03 [0.64, 1.42]
Wasko, 2019 .2	40.9	25.3	76.59	19.87	1.54 [1.37, 1.71]
Boje, 2019	42.76	22.92	69.49	25.48	1.10 [0.96, 1.24]
RE Model for Subgroup					1.24 [0.92, 1.57]

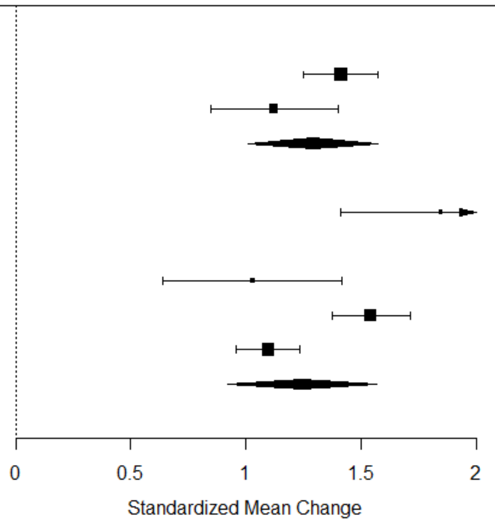

Forest plot measuring change in **Sport and Recreation** subscale score following periacetabular osteotomy
Abbreviations: RE, random effects model; SD, standard deviation

Supplementary appendix 9:

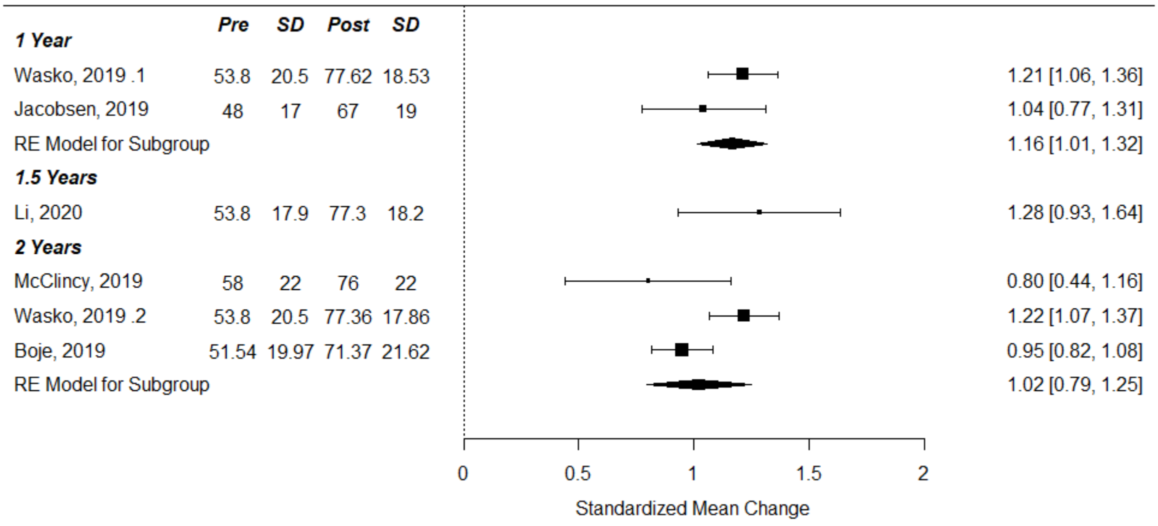

Forest plot measuring change in **Symptoms** subscale score following periacetabular osteotomy
Abbreviations: RE, random effects model; SD, standard deviation

Supplementary appendix 10:

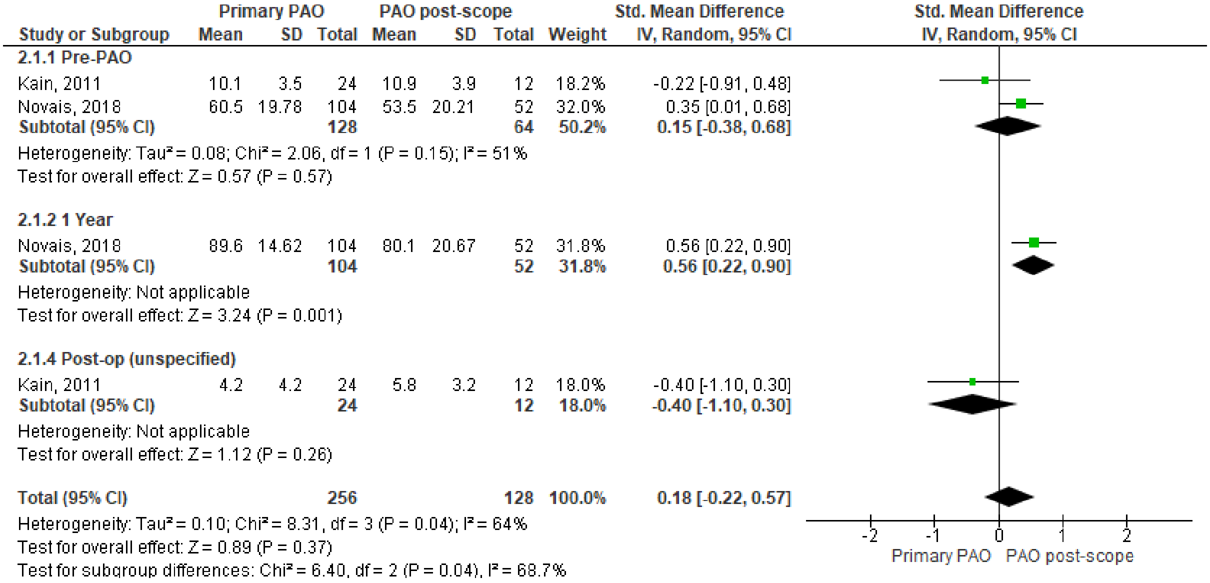

Forest plot comparing **Pain** subscale scores in those undergoing primary PAO and those undertaking PAO following previous arthroscopy.
Abbreviations: CI, confidence interval; IV, Random, random effects model; Std, standardised; SD, standard deviation; PAO, periacetabular osteotomy

Supplementary appendix 11:

Study or Subgroup	Primary PAO Mean	Primary PAO SD	Primary PAO Total	PAO post-scope Mean	PAO post-scope SD	PAO post-scope Total	Weight	Std. Mean Difference IV, Random, 95% CI
**2.2.1 Pre-PAO**								
Kain, 2011	2.6	2	24	4.1	1.7	12	18.7%	-0.77 [-1.49, -0.05]
Novais, 2018	58.6	23.84	104	57	26.13	52	31.1%	0.06 [-0.27, 0.40]
**Subtotal (95% CI)**			**128**			**64**	**49.8%**	**-0.29 [-1.10, 0.52]**
Heterogeneity: Tau² = 0.27; Chi² = 4.26, df = 1 (P = 0.04); I² = 77%								
Test for overall effect: Z = 0.70 (P = 0.48)								
**2.2.2 1year post-PAO**								
Novais, 2018	83.8	16.6	104	76.8	20.79	52	31.0%	0.38 [0.05, 0.72]
**Subtotal (95% CI)**			**104**			**52**	**31.0%**	**0.38 [0.05, 0.72]**
Heterogeneity: Not applicable								
Test for overall effect: Z = 2.25 (P = 0.02)								
**2.2.4 Post-op (unspecified)**								
Kain, 2011	1.9	2.1	24	2.6	1.2	12	19.2%	-0.37 [-1.07, 0.33]
**Subtotal (95% CI)**			**24**			**12**	**19.2%**	**-0.37 [-1.07, 0.33]**
Heterogeneity: Not applicable								
Test for overall effect: Z = 1.03 (P = 0.30)								
**Total (95% CI)**			**256**			**128**	**100.0%**	**-0.08 [-0.51, 0.36]**
Heterogeneity: Tau² = 0.13; Chi² = 10.13, df = 3 (P = 0.02); I² = 70%								
Test for overall effect: Z = 0.34 (P = 0.74)								
Test for subgroup differences: Chi² = 5.09, df = 2 (P = 0.08), I² = 60.7%								

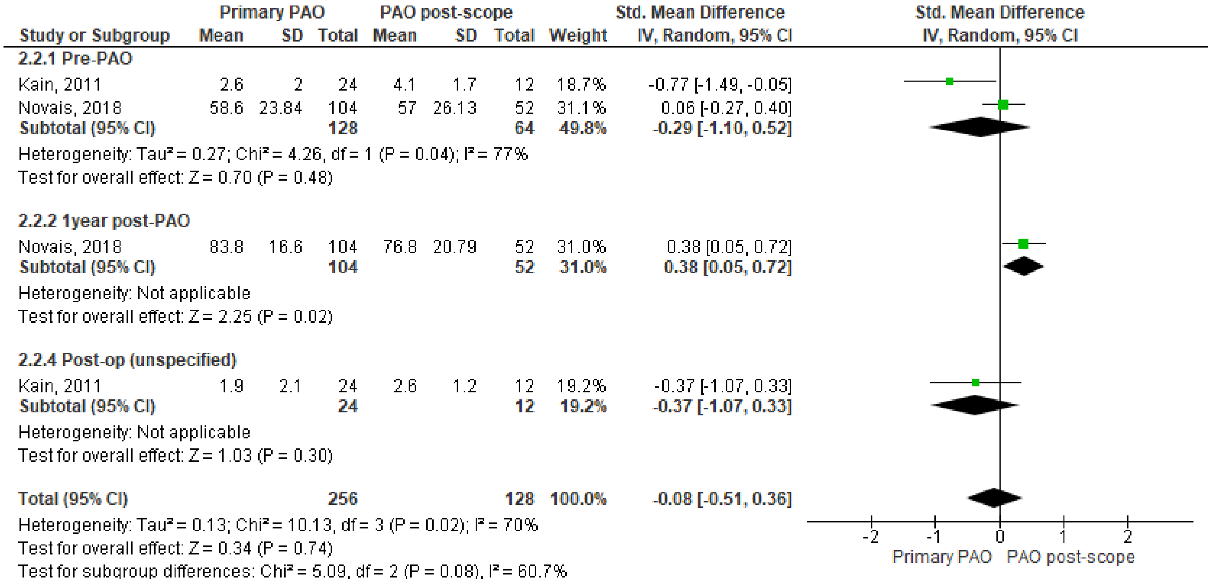

Forest plot comparing **Stiffness** subscale scores in those undergoing primary PAO and those undertaking PAO following previous arthroscopy.
Abbreviations: CI, confidence interval; IV, Random, random effects model; Std, standardised; SD, standard deviation; PAO, periacetabular osteotomy

Supplementary appendix 12:

Study or Subgroup	Primary PAO Mean	Primary PAO SD	Primary PAO Total	PAO post-scope Mean	PAO post-scope SD	PAO post-scope Total	Weight	Std. Mean Difference IV, Random, 95% CI
**2.3.1 Pre-PAO**								
Kain, 2011	22	11.1	24	29	16	12	19.4%	-0.53 [-1.24, 0.17]
Novais, 2018	68.6	18.92	104	61.1	23.47	52	30.6%	0.36 [0.03, 0.70]
**Subtotal (95% CI)**			**128**			**64**	**50.0%**	**-0.03 [-0.90, 0.84]**
Heterogeneity: Tau² = 0.32; Chi² = 5.04, df = 1 (P = 0.02); I² = 80%								
Test for overall effect: Z = 0.06 (P = 0.95)								
**2.3.2 1year post-PAO**								
Novais, 2018	91.9	13.02	104	85.5	17.43	52	30.6%	0.44 [0.10, 0.77]
**Subtotal (95% CI)**			**104**			**52**	**30.6%**	**0.44 [0.10, 0.77]**
Heterogeneity: Not applicable								
Test for overall effect: Z = 2.54 (P = 0.01)								
**2.3.4 Post-PAO (unspecified)**								
Kain, 2011	10.6	13.2	24	17.7	14.3	12	19.4%	-0.51 [-1.22, 0.19]
**Subtotal (95% CI)**			**24**			**12**	**19.4%**	**-0.51 [-1.22, 0.19]**
Heterogeneity: Not applicable								
Test for overall effect: Z = 1.42 (P = 0.15)								
**Total (95% CI)**			**256**			**128**	**100.0%**	**0.04 [-0.41, 0.49]**
Heterogeneity: Tau² = 0.14; Chi² = 10.78, df = 3 (P = 0.01); I² = 72%								
Test for overall effect: Z = 0.18 (P = 0.85)								
Test for subgroup differences: Chi² = 6.03, df = 2 (P = 0.05), I² = 66.8%								

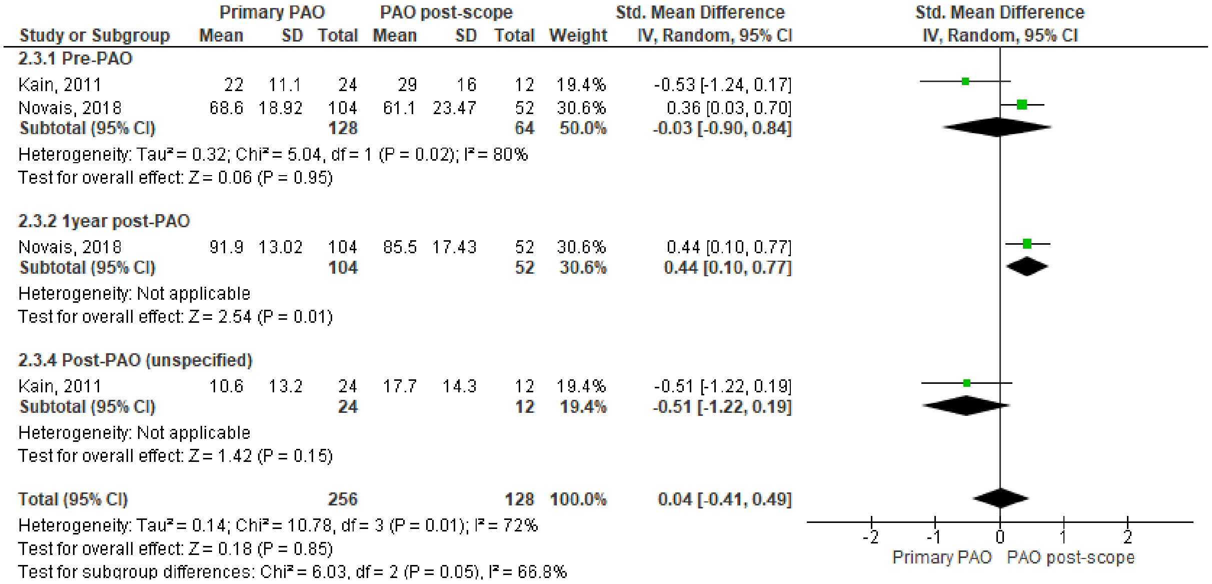

Forest plot comparing **Function** subscale scores in those undergoing primary PAO and those undertaking PAO following previous arthroscopy.
Abbreviations: CI, confidence interval; IV, Random, random effects model; Std, standardised; SD, standard deviation; PAO, periacetabular osteotomy
